# The Role of Chemokines in Orchestrating the Immune Response to Pancreatic Ductal Adenocarcinoma

**DOI:** 10.3390/cancers16030559

**Published:** 2024-01-28

**Authors:** Alexander A. Lekan, Louis M. Weiner

**Affiliations:** Department of Oncology, Georgetown Lombardi Comprehensive Cancer Center, Georgetown University Medical Center, 3970 Reservoir Road NW, Washington, DC 20057, USA; aal75@georgetown.edu

**Keywords:** chemokine, pancreatic ductal adenocarcinoma (PDAC), tumor microenvironment (TME), cancer-associated fibroblasts (CAFs), immunotherapy, T cell, natural killer (NK) cells, tumor-associated macrophages (TAMs)

## Abstract

**Simple Summary:**

Pancreatic ductal adenocarcinoma (PDAC) has a dismal 5-year survival rate of only 11.6%, partially due to limited therapeutic options. Immunotherapy-based approaches, such as immune checkpoint inhibitors, have proven ineffective, in part due to the inability of cytotoxic, effector immune cells to sufficiently infiltrate tumors. Thus, understanding how the PDAC tumor microenvironment (TME) regulates the accumulation of immune cells is critical to improving immunotherapy-based approaches.

**Abstract:**

Chemokines are small molecules that function as chemotactic factors which regulate the migration, infiltration, and accumulation of immune cells. Here, we comprehensively assess the structural and functional role of chemokines, examine the effects of chemokines that are present in the pancreatic ductal adenocarcinoma (PDAC) tumor microenvironment (TME), specifically those produced by cancer cells and stromal components, and evaluate their impact on immune cell trafficking, both in promoting and suppressing anti-tumor responses. We further explore the impact of chemokines on patient outcomes in PDAC and their role in the context of immunotherapy treatments, and review clinical trials that have targeted chemokine receptors and ligands in the treatment of PDAC. Lastly, we highlight potential strategies that can be utilized to harness chemokines in order to increase cytotoxic immune cell infiltration and the anti-tumor effects of immunotherapy.

## 1. Introduction

Pancreatic ductal adenocarcinoma (PDAC) accounts for approximately 90% of all diagnosed pancreatic neoplasms in the United States [1,2]. Currently, the five-year survival rate for PDAC is about 11.6%, an increase of only 5% since the year 2000 and among the worst of all major solid tumors [1]. By 2030, it is projected to become the second leading cause of cancer death, behind only lung cancer [2]. Cancer immunotherapy, with the advent of immune checkpoint inhibitors (ICIs) and cellular therapies, has revolutionized the treatment of cancer in multiple tumor indications [3,4]. Results have been underwhelming in PDAC, however, with virtually all trials testing ICIs failing. Notably, the response to ICI immunotherapy is highly associated with an increase in the proportion of activated intra-tumoral immune cells, particularly CD8^+^ T cells [5,6]. Due to poor immune infiltration and a non-inflamed phenotype, PDAC is considered a “cold” tumor that is refractory to immunotherapy treatment [7,8]. The primary contributor to this phenomenon is PDAC’s tumor microenvironment (TME), the hallmark of which is a dense desmoplastic stroma, made up of cancer-associated fibroblasts (CAFs). The PDAC TME is a complex network of cells, vessels, and molecules, along with the associated signaling pathways, all of which directly or indirectly contribute to PDAC progression. Critically, in PDAC, the TME contributes to the lack of activated immune cell infiltration through the misdirection and sequestration of immune cells, inactivating them before they are able to reach their target (i.e., malignant epithelial cells) and limiting their anti-tumor activity [9,10]. Given that studies have found a correlation between the increased intra-tumoral expression of T and natural killer (NK) cell markers and improved outcomes and increases in patient survival, finding ways to increase activated immune cell infiltration in PDAC, particularly near malignant epithelial cells, is an appealing approach [11,12]. Since immune cell trafficking is driven primarily by chemokines, potentially manipulating these molecules can be utilized to achieve this goal.

Chemokines are small molecules that signal through cell-surface G-protein-coupled receptors that are pleotropic in their function [13]. First identified as chemotactic agents in 1987, with the characterization of IL-8 (now called CXCL8), chemokines primarily function to create gradients to influence the migration of immune cells, as well as other cells such as epithelial and endothelial cells [14]. In the PDAC TME, chemokines can be expressed by a plethora of cells, including cancer cells, CAFs, and both effector immune cells, such as NK cells, and immunosuppressive cells, such as myeloid-derived suppressor cells (MDSCs) and T regulatory cells (Tregs) [15]. In addition to cell recruitment and immune activation, chemokines play important roles in physiological processes, such as morphogenesis and wound healing, and pathological processes, such as cancer metastasis and proliferation [16,17,18]. Furthermore, given that most chemokine receptors are non-specific and have multiple chemokines as ligands, there is a high degree of redundancy and pathway overlap [13]. Therefore, chemokines can directly and indirectly affect tumor immunity, shape immune responses, and influence cancer therapy and outcomes. Significantly, given that chemokines are pleotropic, the same chemokine can recruit either activating or suppressive immune cell types based on the spatial context in which it is present [19,20].

The infiltration of immune cells, driven by chemokines, is a key factor in PDAC prognosis [21]. Critically, the migration of immune cells into the PDAC TME varies significantly due to its heterogenous nature. Thus, understanding the specific spatial architecture of the chemotactic environment in the PDAC TME is crucial to identifying chemokines both critical and detrimental to the efficacy of immune-modulating therapies, such as ICIs. Additionally, better understanding the immune- or tumor-promoting roles of the chemokine network in PDAC is essential for finding ways to overcome immunotherapy resistance. To better understand how chemokines affect the immune response in PDAC, here, I provide a comprehensive overview chemokine structure and function, the roles of chemokines in immune cell infiltration and in PDAC patient outcomes, how chemokines interact with immunotherapies, and current treatment strategies targeting and utilizing chemokines in their approaches.

## 2. Chemokine Structure and Function

**Figure 1:**
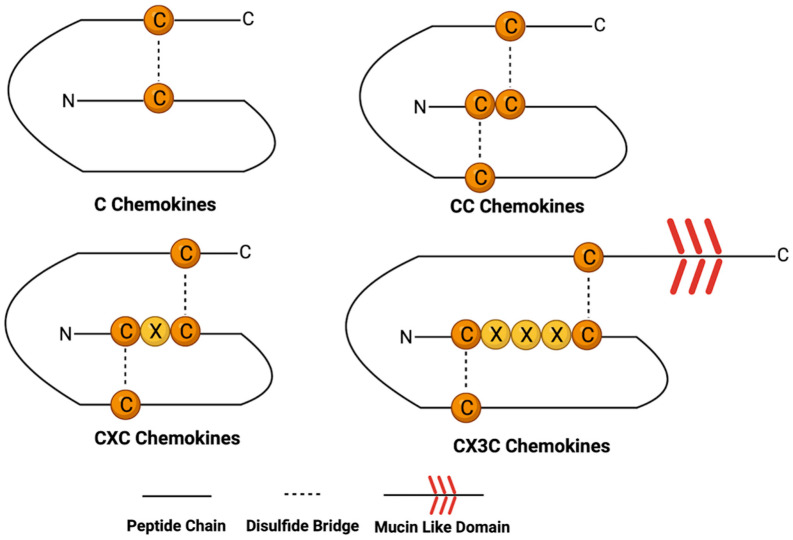
Schematic diagrams of variations in chemokine structure between four subfamilies. Diagrams highlight the variation in number of cysteines (orange circle) between the subfamilies, as well as presence of non-specific amino acids (yellow circle) present between N-terminus cysteines in CXC and CX3C chemokines. Note presence of mucin-like domain in CX3C chemokines, allowing for surface adhesion and existence as a membrane-bound chemokine [created with BioRender.com, accessed on 14 July 2023].

Chemokines are a group of small-molecular-weight (ranging from 8–12 kilodaltons), structurally related polypeptides that regulate the chemotactic activity of cells. To date, approximately 45 chemokines have been definitively identified in humans [22]. The similarity in the gene sequence and amino acid homology between chemokines varies from less than 20% to over 90% of variation between some [13]. Usually, most chemokines are produced as pro-peptides, with a signal recognition peptide of about 20 amino acids which is cleaved during the process of secretion. Chemokines all possess conserved amino acids essential for creating their tertiary structures, defined by four invariant cysteine residues that form disulfide bonds. Often, the first cysteine forms a covalent bond with the third and the second with the fourth [13]. The first two cysteines are found close to the N-terminus of the protein while the third resides close to the center of the molecule and the fourth near the C-terminus [13,22,23]. Additionally, chemokines contain three β-sheets, which are arranged in the shape of a Greek key, overlaid by a *C*-terminal α-helical domain and flanked by an *N*-terminal domain lacking order [13]. Based on the presence of and spacing between the two cysteine residues, chemokines can further be broken down into four classes, C, CC, CXC, and CX3C (where X is any amino acid), as seen in Figure 1.

### 2.1. C Chemokines

C chemokines (or γ-chemokines) only have two cysteine residues, one at the N-terminus and one downstream, unlike all other chemokines. This chemokine family is composed of only two known members, XCL1 and XCL2, where both interact with the XCR1 receptor [24]. Both chemokines have almost identical tertiary structures and are inflammatory chemokines secreted by activated T and NK cells [24]. While initially thought of as mediating NK- and T-cell chemotaxis, XCL1/2 are now believed to be critical for mediating interactions between antigen-presenting cells, especially dendritic cells, and T cells [25,26,27]. With regard to pancreatic cancer, Burrack et al. identified XCR1 signaling, promoted by XCL1, as a driver of type 1 dendritic cell accumulation, which was essential for T-cell anti-tumor effects seen from ICI blockade or CD40 agonism therapy [28].

### 2.2. CC Chemokines

CC chemokines (or β-chemokines) have four cysteine residues, two of which are adjacent near the N-terminus, forming two disulfide bridges. This chemokine family is the largest, with 27 distinct members (CCL1-28; CCL9 and CCL10 are identical) of the subgroup signaling through 10 distinct receptors (CCR1-10) that have been identified to date [24]. Given this disparity in chemokines to chemokine receptors, multiple CC chemokines usually signal through a single receptor. CC chemokines play a crucial role in the function of immune cells, inducing signaling in both lymphocytes, such as T and NK cells, and myeloid-derived immune cells, such as neutrophils, eosinophils, and basophils [29,30]. While contributing to inflammation and anti-tumor effects, through the accumulation of effector immune cells, CC chemokines also harbor multiple pro-tumorigenic functions, including recruiting supporting cells, such as MDSCs and Tregs, and increasing the proliferation, migration, and invasiveness of cancer cells [31,32]. Very often, if not always, a CC chemokine can exhibit both pro-cancer and anti-cancer effects. With regard to pancreatic cancer, multiple CC chemokines have been implicated as both anti- and pro-tumorigenic. For example, Kalbasi et al. identified CCL2 as being produced by orthotopically implanted Kras^LSL-G12D/+^, Trp53^LSL-R172H/+^, and Pdx-1 Cre (KPC) PDAC tumors after treatment with radiotherapy to recruit tumor-associated macrophages (TAMs) and promote tumor growth [33]. Alternatively, Huffman et al. demonstrate that CCL5 is produced by intra-tumoral myeloid cells in KPC tumors in response to CD40 agonism and is crucial for the influx of CD4^+^ T cells and anti-tumor effects seen with immunotherapy [34].

### 2.3. CXC Chemokines

CXC chemokines (or α-chemokines) also have four cysteine residues, similar to CC chemokines. The difference lies in the fact that the two cysteines near the N-terminus are separated by one amino acid (represented in the name with an “X”). CXC chemokines are the second largest family of chemokines, with 17 distinct members (CXCL1-17) of the subgroup signaling through 7 distinct receptors (CXCR1-7) that have been identified to date [24,35]. The CXC family can be further subdivided based on the presence or absence of a glu-leu-arg (ELR) amino acid motif that immediately precedes the first cysteine residue in certain CXC chemokines [35]. CXC chemokines that have the ELR motif (ELR^+^ CXC) have significant neutrophil and monocyte chemotactic and activating characteristics and are potent promoters of angiogenesis [35,36]. Most CXC chemokines have this motif. The CXC chemokines that are negative for it (ELR^−^ CXC), which are termed the interferon (IFN)-inducible CXC chemokines, include CXCL4, CXCL9, CXCL10, CXCL11, and CXCL12, and are strongly chemotactic for activated/memory T and NK cells [37]. These ELR^−^ CXC chemokines are also potent inhibitors of angiogenesis, except for CXCL12 which is pro-angiogenic [38]. Similar to the CC family of chemokines, CXC chemokines also have multiple anti- and pro-tumorigenic effects. With regard to pancreatic cancer, Zhang et al. highlighted how CXCL8 promoted murine TAM tracking in pancreatic cancer tumors, limiting the efficacy of anti-programmed death 1 (anti-PD1) ICI therapy [39]. Treatment with IFN-γ inhibited the CXCL8 released, diminishing TAM trafficking and enhancing ICI anti-tumor effects [39]. Alternatively, Fitzgerald et al. demonstrated that increasing the intra-tumoral levels of CXCL9/10 in murine, KPC-derived PDAC tumors increased the recruitment of CXCR3^+^ NK and T cells and induced anti-tumor effects [12].

### 2.4. CX3C Chemokines

The CX3C chemokine (or d-chemokines) family only consists of one known member, CX3CL1, interacting with one receptor, CX3CR1. Similar to CC and CXC chemokines, CX3CL1 also has four cysteine residues but with three amino acids between the two cysteines near the N-terminus. CX3CL1 is unique, acting as a transmembrane protein and existing in two forms (either membrane-attached or soluble) [40,41]. The soluble form shed from membranes is strongly chemotactic for T cells, NK cells, and monocytes, while, when cell-bound, the chemokine promotes the adhesion of leukocytes to endothelial cells [41,42]. Given its potent leukocyte-signaling abilities, it is classified as a pro-inflammatory chemokine, but has also been implicated in having tumor-promoting roles [43,44]. With regard to pancreatic cancer, Marchesi et al. identified that the CX3CR1 expression on neoplastic pancreatic cancer cells contributes to the perineural invasion of PDAC epithelial cells, given that neurons and nerve fibers express CX3CL1 [45]. Additionally, Celesti et al. examined the CX3CR1 expression in 104 human PDAC and pancreatic intraepithelial neoplasia (PanIN) samples and found that the expression was involved in the early progression from PanIN to PDAC [46].

### 2.5. Chemokine Function

Chemokines induce migration by binding to chemokine receptors, which are G-protein-coupled receptors. Chemokine receptors are embedded in the lipid bilayer of the cell surface and possess seven transmembrane domains [13]. Upon the binding of the chemokine to the receptor, a conformational change is induced, activating signaling pathways and promoting migration. The activation of the chemokine receptor, and subsequent signaling, occurs in two steps, where, first, the main body of the chemokine recognizes and binds the receptor, followed by a conformational change in the chemokine in the second step [13,17]. This conformational change is critical in order to allow the receptor activation and subsequent signaling. Common to all chemokine receptors, receptor stimulation leads to the GDP/GTP exchange of the coupled heterotrimeric G_i_ proteins and the subsequent dissociation of the βγ subunits, leading to the activation of phosphoinositide-specific phospholipase Cβ (PLC) and phosphoinositide 3-kinase (PI3K) [47]. PLC produces inositol-trisphosphate (IP_3_) and diacylglycerol (DAG), which subsequently trigger calcium mobilization and the activation of protein kinase C (PKC), respectively [47]. PI3K generates 3-phosphoinositides, which act as anchors in recruiting proteins with pleckstrin homology domains to the membrane [47]. The further activation of downstream signaling domains differs based on the chemokine receptor.

In addition to inducing the migration of cells in subset-specific manners, chemokines also increase the expression and binding avidity of integrin receptors on the surface of cells [19]. This is accomplished through inside-out signaling pathways [48]. Integrin upregulation is essential for circulating leukocytes to be arrested on endothelial surfaces, supporting trans-endothelial migration, and lymphocyte homing [48,49]. Therefore, this function of chemokines is critical for the accumulation of leukocytes. In relation to the β2 integrins, Mac-1 and LFA-1, which are highly expressed on leukocytes, chemokines from both the CC and CXC families have been shown to trigger adhesion via these integrins across a variety of cell types, including neutrophils, eosinophils, lymphocytes, and monocytes [49,50,51,52]. Thus, chemokines help facilitate the accumulation of leukocytes to specific locations not only by creating gradients, but also through increasing cellular adhesion to endothelial cell walls, a dual-pronged approach helping to ensure the localization of leukocytes in the desired locations.

## 3. Chemokines Present in the PDAC Tumor Microenvironment

Pancreatic tumors exhibit great heterogeneity in the cellular composition, the vasculature, the extent of extracellular matrix (ECM) deposition, and the types of immune cell infiltrate. These cell-to-cell interactions in the PDAC TME are governed by the secretion of soluble factors such as cytokines and chemokines. In the PDAC TME, several chemokines are secreted by various cell types and contribute to therapeutic resistance and cancer progression. Specifically, chemokines regulate not only immune cell infiltration, but also the cross-talk that occurs between malignant epithelial cells and stromal cells, such as TAMs and CAFs. Thus, this signaling network allows tumors to grow and evolve in response to therapeutic and immune pressures, such as chemotherapy, radiotherapy, and immunotherapy treatments.

The PDAC TME produces a wide variety of chemokines. These chemokines are produced not only by cancer cells, but also by TAMs, CAFs, MDSCs, and structural support elements, such as endothelial cells. Critical to understanding the function of these chemokines, in the context of PDAC, however, is which cells are producing them and where they are localized in the PDAC TME.

### 3.1. Chemokines Produced by Malignant Epithelial Cells

**Figure 2:**
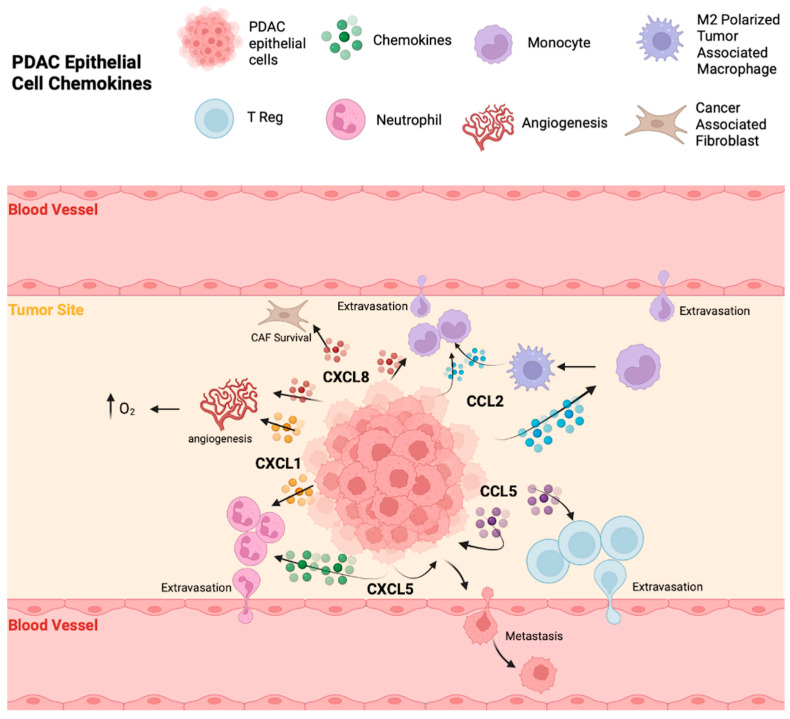
Simplified overview of how chemokines elaborated by malignant epithelial cells in PDAC promote tumorigenesis through a variety of mechanisms. Chemokines elaborated by malignant epithelial cells promote accumulation of immunosuppressive cells, such as Tregs, TAMs, and TANs, induce angiogenesis, and aid in survival of CAFs [created with BioRender.com, accessed on 14 July 2023].

Malignant epithelial cells in PDAC express a variety of chemokines and chemokine receptors in order to promote a beneficial TME for themselves. Figure 2 demonstrates the roles of major chemokines produced by malignant epithelial cells in PDAC.

#### 3.1.1. CCL2

Pancreatic tumor cells begin secreting CCL2 soon after malignant transformation [53]. CCL2 is a potent chemoattractant for monocytes and macrophages, signaling via the CCR2 and CCR4 cell surface receptors, and is vital for attracting TAMs and inducing tumorigenesis [54]. After the transformation of monocytes into TAMs, via various immunosuppressive factors produced by the PDAC TME such as transforming growth factor-β (TGF-β) and IL-10, these TAMs can then begin to secrete CCL2 themselves, creating an amplification loop [55]. CCL2 can additionally be expressed by CAFs in later stages of tumor growth and formation [56]. Interestingly, CCL2 appears critical to orchestrating the immunosuppressive PDAC TME, and its production is in part induced by mutations in KRAS. Liu et al. demonstrated that, in response to peroxisome-proliferator-activated receptor-delta (PPARδ), a lipid nuclear receptor upregulated in PanINs, ligand activation induced mutant KRAS epithelial cells to secrete CCL2 [57]. This drove the creation of an immunosuppressive TME and PanIN progression to PDAC [57]. Further providing evidence that malignant epithelial cells produce CCL2, and not just stromal or immune components, Kalabasi et al. found that intra-tumoral CCL2 levels of orthotopically implanted KPC tumors increased in response to radiotherapy, as well as CCL2 expression in PDAC cell lines in vitro after treatment with radiotherapy [33]. Thus, CCL2 appears not only critical for PDAC progression, but is also produced in response to stressors directed against the malignant epithelial cells, such as radiotherapy. While CCL2 indirectly benefits pancreatic cancer, through fostering the creation of an immunosuppressive TME, CCL2 can also directly act on cancer cells in an autocrine fashion. It has been shown in glioma and breast cancer cell lines that CCL2 expression and receptor engagement can increase proliferation and cancer stem cell self-renewal, suggesting direct roles that may benefit tumor cells in PDAC as well [58].

#### 3.1.2. CCL5

CCL5, also known RANTES (regulated on activation, normal T cell expressed and secreted), is a potent chemoattractant for a variety of leukocytes, including T cells, monocytes, NK cells, and basophils, signaling via the CCR1, CCR3, and CCR5 cell surface receptors [59]. Among these receptors, CCL5 has the highest affinity for CCR5. CCL5 exerts pro-tumorigenic effects in PDAC by both acting on PDAC epithelial cells and recruiting beneficial cells to the PDAC TME. Given its role as a potent chemoattractant for T cells, CCL5 can be utilized to attract Tregs to malignant epithelial cells. Wang et al. demonstrated that Forkheadbox protein 3 (FOXP3), a key transcription factor for Tregs, was highly expressed in pancreatic cancer cell lines, which, in turn, upregulated CCL5 expression [60]. CCL5 expression increased the recruitment of Tregs, in vitro and in vivo, which could be ablated with a blockade of CCL5. Further work by Wang et al. demonstrated that FOXP3 expression additionally increased programmed death ligand 1 (PD-L1) expression, which, when inhibited with CCL5, decreased the tumor burden and Treg infiltration in orthotopic murine, Pan-02 PDAC tumors [61]. With regard to CCL5’s pro-tumorigenic functions on PDAC epithelial cells, Singh et al. identified the increased expression of CCL5/CCR5 in metastatic pancreatic cancer tissues, via immunohistochemistry (IHC), as compared to the non-neoplastic kind [62]. The treatment of pancreatic cancer cell lines, which all expressed CCR5, with CCL5 increased the invasive potential and induced the proliferation of cells via F-actin polymerization [62]. This suggests CCL5 not only remodels the PDAC TME to benefit tumor cells, but can also enhance the tumor cell’s metastatic potential. Similar to CCL2, however, CCL5 expression is not exclusive to pancreatic cancer cells, especially as PDAC progresses. As previously mentioned, Huffman et al. demonstrated that CCL5 is primarily produced by intra-tumoral myeloid-derived cells in orthotopic KPC tumors [34]. Additionally, Makinoshima et al. highlight how the co-culture of pancreatic cancer cells with mesenchymal stromal cells (MSCs) induces the MSC production of CCL5 [63]. Importantly, however, they showcase that pancreatic cancer cells can express CCL5 prior to co-culture [63]. Thus, while support cells in the PDAC TME, such as myeloid cells and TAMs, can express CCL5 later on, pancreatic cancer cells’ ability and early expression of the chemokine lead to classifying it as produced by malignant epithelial cells.

#### 3.1.3. CXCL1

CXCL1 is part of the ELR^+^ CXC chemokines and, thus, is a potent chemoattractant of neutrophils, signaling through the CXCR2 receptor. Additionally, it has a role in angiogenesis and is involved in the act of PDAC progression [24]. CXCL1 plays a multifaceted role in PDAC progression. Matsuo et al. highlighted how, even though multiple pancreatic cancer cell lines expressed CXCL1, this did not increase their proliferation in an autocrine fashion [64]. Instead, CXCL1 played a role in promoting angiogenesis in a paracrine manner in human vascular endothelial cells (HUVECs), and blocking CXCR2 in an orthotopic murine pancreatic cancer model reduced the tumor volume and inhibited the microvessel density [64]. Additionally, Niu et al. demonstrate that the tumor cell intrinsic loss of SETD2 in pancreatic cancer cells boosted the PI3K signaling and expression of CXCL1, promoting neutrophil recruitment and immunosuppression [65]. Significantly, the treatment of orthotopic Pdx^cre^, LSL-Kras^G12D^, and Setd2^f/f^ (KSC) tumors, with a CXCR2 antagonist reduced tumor-infiltrating neutrophiles, but had much less of an effect on tumor-infiltrating macrophages and monocytes [65]. This highlights how CXCL1’s role, converse to CCL2, is to promote angiogenesis and neutrophil recruitment, with less of a focus on TAM recruitment. Highlighting CXCL1’s role in neutrophil recruitment by pancreatic cancer cells, Bianchi et al. identified CXCL1 as a key mediator of spatial T-cell restriction due to CXCR2^+^ neutrophils in human PDAC using imaging mass cytometry (IMC) [66]. The neutrophil-produced tumor necrosis factor (TNF) induces feed-forward CXCL1 overproduction in both tumor cells and CAFs, leading to T-cell suppression [66]. Thus, CXCL1’s roles in angiogenesis and neutrophil attraction highlight how tumor cells utilize it in order to remake the PDAC TME to their advantage.

#### 3.1.4. CXCL5

CXCL5 is another ELR^+^ CXC chemokine and, thus, also potently attracts neutrophils. Just like CXCL1, CXCL5 also signals through CXCR2, explaining why, often, CXCL5 and CXCL1 are seen to function in parallel in PDAC. With regard to neutrophil chemotaxis, Deng et al. identified that CXCL5 expression in pancreatic cancer cell lines can be induced by discoid domain receptor (DDR) 1 signaling, which is a tyrosine kinase receptor specifically activated by fibrillar collagens [67]. The activation of DDR1 by collagen enhanced the CXCL5 expression in orthotopically injected MDA-PATC 148 pancreatic cancer cells, which induced tumor-associated neutrophil (TAN) accumulation and neutrophil extracellular trap (NET) formation [67]. Critically, CXCL5 production and TAN accumulation helped promote cancer cell invasion in in vitro assays [67]. Similarly, Wang et al. identified that CXCL5 was increased in human pancreatic tissue compared to the normal pancreas, and the knockdown of CXCL5 in pancreatic cancer cell lines reduced the proliferation and migration ability of cells [68]. Critically, knockdown significantly decreased the growth of xenograft tumors in vivo, suggesting CXCL5 expression by pancreatic cancer cells is necessary not only for metastasis, but also for optimal cell proliferation [68]. Further supporting that CXCL5 is secreted by epithelial pancreatic cancer cells, Chao et al., using tumor-bearing KPC and KPC with the Rosa^LSL-YFP^-allele (KPCY) mice, found that the CXCL5 expression was primarily concentrated in the tumor as opposed to the stroma [69]. While stromal cells elaborated multiple CXCR2 ligands, CXCL5 was mainly produced by pancreatic cancer cells and its expression was linked with mutant KRAS status [69]. Interestingly, pancreatic cancer cells also appear to upregulate CXCL5 in response to gemcitabine chemotherapy, similar to how CCL2 is upregulated in response to radiotherapy. Lee et al. demonstrated that CXCL5 was critical to pancreatic cancer cell resistance to gemcitabine in vivo, where the knockdown of CXCL5 enhanced the inhibitory anti-tumor effects of treatment and promoted apoptosis [70]. Thus, while CXCL5 appears to have multiple positive effects acting as a chemoattractant to promote PDAC tumorigenesis, it also appears to function in an autocrine manner, increasing pancreatic cancer cell fitness and viability.

#### 3.1.5. CXCL8

As previously mentioned, CXCL8 was the first chemokine discovered in 1987. Since its discovery, it has been found to be produced by a variety of cell types, including immune cells, epithelial cells, and fibroblasts. CXCL8 is also an ELR^+^ CXC chemokine and plays a vital role in the pro-inflammatory signaling pathway, acting as the primary chemokine involved in the recruitment of neutrophils. It exerts its chemotactic effects via interactions with the CXCR1 and CXCR2 receptors. Importantly, CXCL8 has been found to be constitutively expressed in a variety of solid tumors, including melanoma, glioma, and colon and pancreatic cancer [71,72,73]. While constitutively expressed by a plethora of pancreatic cancer cell lines, CXCL8 is interesting in that its secretion by cancer cells is greatly upregulated due to interactions between pancreatic cancer cells and other cell types in the PDAC TME. Awaji et al. highlight that CAFs, via the secretion of fibroblast growth factor (FGF) 2, induced pancreatic tumor cells to increase the expression of CXCL8 in an in vitro co-culture system [74]. Significantly, CXCL8 secretion by the tumor cells stimulated and maintained the survival of CAFs, highlighting its beneficial role to pancreatic cancer cells [74]. Further supporting this bi-directional, mutually beneficial relationship orchestrated through CXCL8 secretion by tumor cells, Matsuo et al. reported that the CAF production of CXCL12 significantly enhanced the pancreatic cancer cell secretion of CXCL8 [75]. This, in turn, enhanced the proliferation/invasion of HUVECs, but had no effect on pancreatic cancer cell proliferation/invasion [75]. Thus, CXCL8 appears beneficial to pancreatic cancer by promoting the angiogenesis and vascularization of the PDAC TME. In addition to its angiogenic properties, Zhang et al. have also reported that CXCL8 mediates immunosuppression through the attraction of TAMs, which highly express CXCR1 and CXCR2 [39]. In tumor xenograft models, using human pancreatic cancer cell lines, CXCL8 expression was correlated with a preferential expansion of CXCR2^+^CD68^+^ macrophages, which significantly led to the decreased efficacy of anti-PD1 therapy [39]. Critically, treatment with IFN-γ suppressed tumor-derived CXCL8, reducing TAM trafficking and enhancing anti-PD1 efficacy [39]. Thus, not only do TME cells, such as CAFs, signal pancreatic cancer cells to secrete CXCL8 for pro-angiogenic effects, but also increase the accumulation of pro-tumorigenic support cells, such as TAMs, highlighting CXCL8’s nefarious role in promoting PDAC tumorigenesis.

Again, while these chemokines can later be produced in higher quantities by support cells that arrive in the PDAC TME to promote tumorigenesis, it is clear that malignant epithelial cells are key drivers of their elaboration, especially initially, and play a key role in the progression of PDAC.

### 3.2. Chemokines Present in the PDAC Stroma

**Figure 3:**
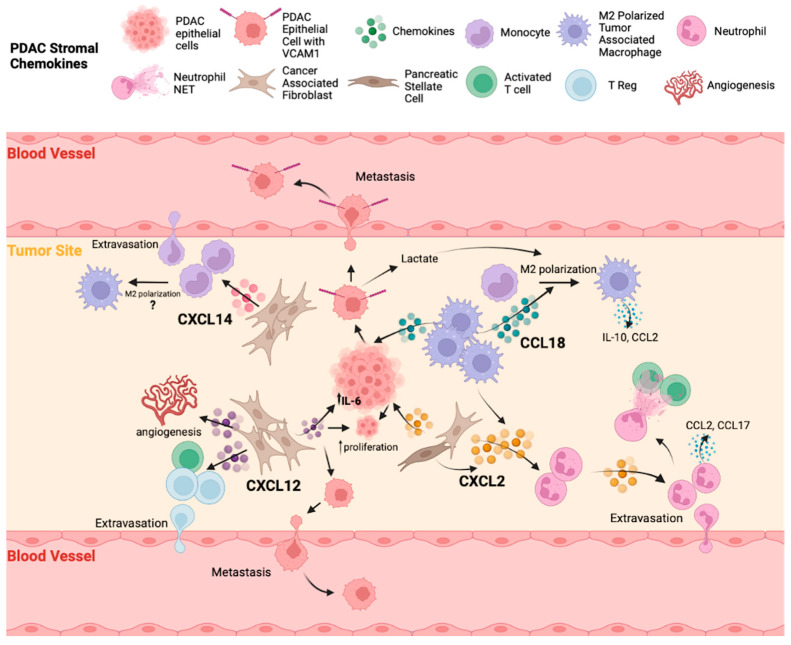
Simplified overview of how chemokines elaborated by cells in the PDAC stroma promote tumorigenesis through a variety of mechanisms. Chemokines elaborated by PDAC stromal cells promote polarization of monocytes to an M2 immunosuppressive phenotype, attraction of TANs to impair activated T-cell attack, Treg accumulation and activated T-cell sequestration, and intra-tumoral angiogenesis. Chemokines also act on PDAC epithelial cells to promote proliferation and metastasis, through mechanisms such as upregulation of VCAM-1, increased lactate production (helping facilitate M2 polarization), and increased IL-6 production [created with BioRender.com, accessed on 14 July 2023].

The pancreatic stroma, composed of multiple cell types, plays a critical role in facilitating tumor progression and growth [10]. While pancreatic cancer cells secrete a variety of chemokines, as already discussed, their expression can be amplified later on by stromal cells, through the creation of positive feedback loops. Yet, a multitude of chemokines are produced almost exclusively by stromal cells, such as TAMs, CAFs, and Tregs, to ensure a beneficial environment for tumor growth that is hostile to therapeutic treatments and immune attack. Figure 3 demonstrates the roles of major chemokines produced by stromal elements in PDAC.

#### 3.2.1. CCL18

CCL18 is produced mainly by antigen-presenting cells of the immune system, such as dendritic cells and macrophages. While CCL18 is a chemokine, it is less known for its chemotactic effects and more for its effect on cells in the PDAC TME, especially TAMs. CCL18 is mainly produced by TAMs in a variety of tumor types, but it plays no role in actually facilitating their initial accumulation [76,77,78]. While CCL18 has recently been discovered to bind and signal through CCR8, it also signals through a variety of non-traditional receptors, with the most critical in neoplastic disease being phosphatidylinositol transfer protein 3 (PITPNM3)/PYK2 N-terminal domain-interacting receptor 1 (Nir1) [79,80]. CCL18 directly and indirectly influence tumorigenesis through a variety of mechanisms, beginning with the polarization of TAMs towards an immunosuppressive, M2 phenotype [81]. Schraufstatter et al. highlighted that the treatment of cultured monocytes with CCL18 induced maturation into macrophages exhibiting an M2 phenotype, which further elaborated immunosuppressive cytokines, such as IL-10, and chemokines such as CCL2, CCL3, CCL22, and CXCL8 by TAMs [81]. Additionally, Su et al. demonstrated, in orthotopic human breast cancer xenografts, that the CCL18 production by TAMs leads to the recruitment of naïve CD4^+^ T cells, via PITPNM3 signaling, which are then induced to differentiate into Tregs [82]. Furthermore, Tregs can then act to increase macrophage M2 polarization and CCL18 production [83]. With regard to pancreatic cancer, Ye et al. demonstrated, in vitro, that the co-culture of TAMs with pancreatic cancer cell lines induced CCL18 expression by TAMs, which led to the upregulation of VCAM-1, an adhesion molecule, in the pancreatic cancer cells [84]. CCL18 was not expressed in pancreatic cancer cells alone. Critically, VCAM-1 upregulation increased the proliferation and migration of cancer cells, and also mediated the recruitment of TAMs to the PDAC TME, facilitating the binding of TAMs to cancer cells [84]. Thus, TAM-secreted CCL18 acted to indirectly facilitate TAM accumulation through VCAM-1 expression. Interestingly, the authors also discovered that VCAM-1 upregulation induced lactate production, which further led to the M2 polarization of TAMs and a positive feedback loop effect [84]. Evaluating the CCL18 expression in human PDAC samples, Meng et al. found that CCL18 was expressed in human PDAC tissue, as analyzed via IHC, and that the expression was higher in mesenchymal (i.e., stromal) cells compared to epithelial cells and that these mesenchymal cells were M2-polarized macrophages [85]. Additionally, they discovered that CCL18 expression by M2-polarized macrophages increased the migratory capability of pancreatic cancer cells in vitro, as assessed via trans-well assays, but, conversely to Ye et al., had no effect on cell proliferation [85]. Additionally, while not a PDAC study, Zeng et al. found that CCL18 signaling from TAMs in in vivo murine breast tumors activated a specific CAF phenotype in normal breast-resident fibroblasts, inducing a chemoresistance phenotype [86]. Given the abundance of CAFs in the PDAC TME, this association is important to note, and it is important to consider that similar events may possibly be taking place.

#### 3.2.2. CXCL2

CXCL2 is also part of the ELR^+^ CXC chemokines, acting as a potent attractant of neutrophils and shown to be responsible for TAN infiltration in multiple tumor types [87]. CXCL2 is known to be produced by monocytes and macrophages as a chemoattractant element for neutrophils [88]. Additionally, Li et al. have shown that neutrophils are able to regulate their own recruitment through the increasing expression of CXCL2, creating a forward feedback loop [89]. While similar to CXCL1 and CXCL5, with its amino acid sequence being ~90% identical to CXCL1 and also signaling through the CXCR2 receptor, its role in PDAC has not been as thoroughly dissected. Chao et al., using tumor-bearing KPCY mice, as previously discussed, found that CXCL2 expression was primarily concentrated in the stromal compartments as opposed to the tumor epithelial compartment using IHC and quantitative polymerase chain reaction (qPCR) [69]. CXCL2 elaboration by stromal cells, such as CAFs, would appear plausible given that Takikawa et al. reported that pancreatic stellate cells (PaSCs), precursors to CAFs, upregulate the secretion of CXCL2 in response to senescence, induced by gemcitabine or hydrogen peroxide [90]. When the conditioned medium taken from these senescent PaSCs was added to cultures of human pancreatic cells lines AsPC-1 and MIA PaCa-2, the proliferation and migration of the cancer cells were increased [90]. These effects were attenuated with the addition of a selective CXCR2 antagonist. Further supporting the stromal production of CXCL2, Shao et al. found that the downregulation of Sequestrome-1, an autophagic substrate and signaling adapter, in cultured PaSCs via short hairpin RNA (shRNA) knockdown led to an inflammatory and senescent phenotype with an upregulated CXCL2 expression, as validated by qPCR [91]. Conversely, Steele et al. reported that, while the CXCL2 expression is also significantly elevated in the KPC mouse model, this expression was primarily seen in the tumor epithelium, as opposed to the stromal compartment [92]. Additionally, they showed that KPC cells in culture also demonstrated the increased production of CXCL2, albeit significantly less than CXCL1 or CXCL5 [92]. Steele et al. also showed that FAP^+^ fibroblasts increased the productions of CXCL2, in addition to CXCL1 and 5 [92]. Supporting Steele’s results, Siolas et al. also reported that gain-of-function p53^R172H^ mutations in Kras^G12D^-mutated mouse pancreatic ductal epithelial cells, orthotopically implanted in immunocompetent mice, led to elevated levels of CXCL5 and CXCL2 production, analyzed via qPCR and multiplex immunoassay [93]. Interestingly, only the knockdown of CXCL2 in cells, via shRNA, reversed neutrophil recruitment and demonstrated fewer intra-tumoral TANs while the knockdown of CXCL5 had no effect on neutrophil recruitment [93]. Yet, Siolas et al. also state that they found CXCL2 expression was significantly higher in the immune compartment as compared to tumor cells, with the opposite being true for CXCL5 [93]. This possibly suggests that, while CXCL5 production is more isolated to tumor cells, CXCL2 appears mostly produced by stromal components, such as infiltrating immune cells. These contradictory reports of CXCL2 production suggest that more research needs to be carried out to fully understand the drivers of CXCL2 production in the PDAC TME. Possibly, CXCL2 production may be initiated by tumor epithelial cells and then enhanced through CAFs and recruited TANs, similar to previously discussed chemokines. Thus, while listed here as a chemokine localized to the PDAC stroma, continuing work may support its inclusion as a chemokine produced by malignant epithelial cells and more concentrated in epithelial compartments.

#### 3.2.3. CXCL12

CXCL12, also known as stromal cell-derived factor 1 (SDF1), is an ELR^−^ CXC chemokine and one of the most widely researched and targeted chemokines in pancreatic cancer. CXCL12 plays multiple vital physiological chemotactic roles, including in embryogenesis, angiogenesis, and hematopoiesis [94]. Given how important it is, it is not surprising that CXCL12 is one of the few chemokines where the knockout (KO) is embryonically lethal in mice [95]. With regard to its pathological roles, CXCL12 is primarily secreted by CAFs and is directly implicated in tumorigenic progression in a variety of tumors, including breast, colon, and pancreatic [96,97,98]. CXCL12 exerts its chemotactic effects by primarily signaling through the CXCR4 receptor [99]. Later work also uncovered that CXCL12 can signal through the CXCR7 receptor [100]. Interestingly, CXCL12 is the only known ligand for CXCR4, unusual given that chemokine receptors usually have multiple ligands, which has been postulated to be an evolutionary redundancy feature [13,94]. This only underscores CXCL12’s essential role in cellular chemotaxis and helps explain its embryonic lethality. Given that CXCR4 is expressed on a range of cell types, from hematopoietic cells to endothelial cells, CXCL12 exerts its effects on a broad range of cells [101]. This is especially relevant in the PDAC TME, where CXCL12 orchestrates a variety of functions. CXCL12, as mentioned, is mainly secreted by CAFs in the PDAC TME. This secretion can be in response to environmental stressors, such as chemotherapy, and, interestingly, can also appear to be in response to stress being exerted on pancreatic cancer cells. Morimoto et al. demonstrated that the treatment of sensitive and resistant human pancreatic cancer cell lines, MIA PaCa-2 and AsPC-1, with gemcitabine chemotherapy increased the expression of CXCR4 in resistant cell lines [102]. Upon co-culturing these gemcitabine-resistant cells with CAFs, the CAF secretion of CXCL12 significantly increased and mediated the invasiveness of these cells in a Matrigel invasion assay system [102]. This increase in CXCR4 by pancreatic cancer cells, leading to increased CXCL12 production by CAFs, has also been documented by others. Interestingly, Zhang et al. found that this increased resistance of pancreatic cancer cells to gemcitabine was mediated by the induction of autocrine IL-6 production, due to CXCL12 [103]. While not examined, given that IL-6 is involved in CAF activation, this suggests that the increased production of IL-6 may be supporting a positive, CXCL12/CAF feedback loop. Supporting the idea that pancreatic epithelial cells rely on CAFs for CXCL12 signaling, Shen et al. found that CXCL12 was not expressed by MIA PaCa-2 cells, as analyzed via qPCR, but CXCR4 was expressed [104]. Additionally, the exogenous addition of CXCL12 to these cells enhanced their proliferation and invasiveness [104]. Apart from direct pro-tumorigenic functions on pancreatic cancer cells, CXCL12 is also heavily involved in mediating the metastasis of pancreatic cancer cells. Xu et al. demonstrated that the expression of CXCL12 by rat dorsal root ganglia (DRG) cells in vitro mediated enhanced interactions between DRG and pancreatic cancer cells (PANC-1 and MIA PaCa-2) [105]. Additionally, an in vivo model, where CXCR4-silenced MIA PaCa-2 cells and control were both injected into the backs of mice, showcased that cells lacking CXCR4 demonstrated less peri-neural invasion [105]. Apart from its chemotactic role in stimulating the invasiveness of cancer cells, Samara et al. also demonstrated that CXCL12 upregulates tumoral matrix metalloprotease (MMP) expression and secretion (MMP-9), leading to the contraction of collagen matrices, in head and neck squamous cell carcinoma [106]. Given that MMP-9 is able to degrade the ECM, which is an essential step in tumor cell extravasation and metastasis, and it is highly expressed in PDAC, CXCL12 may be playing a similar role in upregulating MMPs in PDAC [107]. Interestingly, McQuibban et al. showed how multiple MMPs (including MMP-9) inactivate CXCL12 activity, suggesting that they function as regulatory proteases to attenuate CXCL12 function [108]. Further research is needed to elucidate the interactions between CXCL12 and MMPs, and what role they may be facilitating in promoting metastasis, however, in PDAC.

#### 3.2.4. CXCL14

CXCL14 is an interesting chemokine, in that it is constitutively expressed at high levels in many normal tissues, including adipose, breast, lung, and skin [109]. However, its expression is usually reduced or absent from cancer cells [109]. While more research is required on CXCL14, its multifaceted role in numerous cancers, including PDAC, and its unique structure make it worth discussing. CXCL14 is part of the CXC family of chemokines, yet differs by having a shorter N-terminus and five extra amino acids between its third and fourth cysteines [110]. In terms of chemotactic ability, CXCL14 has been shown to attract monocytes, dendritic cells, and natural killer cells [111,112,113]. Interestingly, CXCL14 does not have an identified receptor; thus, it is not exactly clear how CXCL14 exerts effects on cells. While CXCL14 does not appear to be expressed highly by cancer cells, it has been found to be upregulated in other types of cancer such as pancreatic, breast, and prostate [114,115,116]. In terms of cellular source, the predominant producer of CXCL14 is believed to be fibroblasts. Specifically, in tumors where CXCL14 has been found to be upregulated, the drivers of expression have been reported to be fibroblasts. Sjoberg et al. reported that fibroblasts secreting CXCL14 that were co-cultured with MCF-7, DCIS or SKBR3, all breast cancer cell lines, stimulated lung colonization and increased the metastasis of these cells when injected via the tail vein of 8-week-old SCID mice [117]. Additionally, Augsten et al. reported that CXCL14 is upregulated in CAFs of human prostate cancer via mRNA analysis by qPCR and IHC analysis of protein levels, comparing tumor and matched normal tissue [118]. Interestingly, they also found that the CXCL14-conditioned medium, produced by fibroblasts, had a chemotactic effect on monocytes in a trans-well system, while recombinant CXCL14 alone did not [118]. This suggests that, in addition to enhancing the cancer cell invasive capabilities, CXCL14 can also help facilitate macrophage infiltration in tumors, possibly leading to increased TAM accumulation. With regard to pancreatic cancer, Wente et al. found that CXCL14 was expressed at very low copy levels by PANC-1, T3M4, and Colo357 pancreatic cancer cells (<6 copies/10 k copies cpb) via qPCR analysis as compared to PDAC tissue samples from which RNA was extracted which had a very high expression (>5000 copies/10 k copies cpb) [115]. Normal pancreatic tissue exhibited significantly lower levels of CXCL14 expression, highlighting how PDAC induces CXCL14 production [115]. Furthermore, the treatment of pancreatic cancer cells with recombinant CXCL14 did not increase cell proliferation nor did CXCL14 protect against gemcitabine-induced apoptosis when cells were treated with the IC_50_ concentration of gemcitabine in vitro [115]. CXCL14 did increase the migratory capacity of pancreatic cancer cells in a trans-well in vitro assay, suggesting a role in increasing the invasive capabilities of cancer cells [115]. While previously discussed in another work, in breast and prostate cancer, focusing on CXCL14 and its secretion by fibroblasts suggest that CXCL14 can be involved in CAF-mediated PDAC resistance mechanisms; more work is needed to fully understand CXCL14’s diverse role in the PDAC TME. Highlighting CXCL14’s pleotropic functions, Rivera et al. found that CXCL14 production by myeloid cells in mouse Rip1Tag2 pancreatic neuroendocrine tumors (PNETs) actually sensitized tumors to vascular endothelial growth factor (VEGF) therapy [119]. Interestingly, tumors from mice that did not respond had a lower expression of CXCL14, and a loss of ability to express CXCL14 by CD11b^+^, a marker for monocytes, granulocytes, and NK cells, coincides with a loss of response to VEGF therapy and the return of tumor angiogenesis [119]. Additionally, the authors demonstrated that the blocking of CXCL14 by the antibody led to a lack of response to therapy [119]. Again, while this is a PNET tumor model, and not PDAC, it is interesting to highlight how intra-tumoral myeloid cells can induce the expression of CXCL14 in a beneficial manner, due to its angio-static properties. Thus, it is clear that CXCL14 is a multi-dimensional chemokine whose role in PDAC has to be more thoroughly explored through future research to fully understand what effects it is having in the PDAC TME.

## 4. Chemokines Influencing Immune Cell Accumulation in PDAC

Given that immune cells traffic towards chemokine gradients, the secretion of various chemokines governs immune cell accumulation in the PDAC TME. While certain chemokines produced by PDAC epithelial cells and PDAC stromal elements promote tumorigenesis, chemokines can also promote anti-tumor immune responses through the attraction of cytotoxic T cells, NK cells, and M1 polarized macrophages. Alternatively, as already discussed, chemokine secretion can also promote the accumulation of tumor-promoting immune cells such as M2-polarized TAMs, TANs, and MDSCs. In order to better promote the anti-tumor effects, however, it is critical to understand which chemokines are promoting anti-tumor responses in the PDAC TME, as well as which are contributing to the accumulation of immune cells that promote tumorigenesis.

### 4.1. Promoting Anti-Tumor Immune Responses

#### 4.1.1. CD8^+^ T Cells

CD8^+^ cytotoxic effector T cells are one of the critical cells in anti-tumor responses. CD8^+^ T cells can kill cells that present antigens in the context of major histocompatibility class I (MHC-I) through the release of cytotoxic molecules, such as perforin and granzyme B, along with secreting pro-inflammatory molecules, such as IFN-γ and TNF-α, to promote the immune response [120]. Given their critical role in producing anti-tumor responses in the context of ICI therapy, considerable work has focused on the trafficking of CD8^+^ T cells in the PDAC TME [121,122]. In the context of CD8^+^ T cells, Romero et al. reported that CD8^+^ T-cell infiltration was strongly associated with the increased expression of a set of four chemokines; CCL4, CCL5, CXCL9, and CXCL10. Examining a cohort of 113 primary resected PDAC samples and 107 PDAC liver mets via IHC, chemokine expression, and transactional hallmarks using RNA sequencing (RNA-seq), the authors found that these four chemokines were the only ones that showed a positive and significant correlation with CD8^+^ T-cell infiltration [123]. Interestingly, PDAC tumors that exhibited this four-chemokine signature were linked to a T-cell-inflamed phenotype, with an increased expression of major markers of T-cell activation and inhibition [123]. Critically, the authors did not find an association between the tumor mutational burden and increased T-cell infiltration [123]. This was a very interesting finding given that the tumor mutational burden has been identified as an emerging biomarker marker of the patient’s response to immunotherapy treatments, such as ICI [124]. Thus, this finding showcases how chemokine expression can potentially be used as a more accurate marker to understand whether patients with PDAC have a better chance to respond to immunotherapy. Further elaborating on the role of CXCR3 ligand chemokines, such as CXCL9 and CXCL10, Vonderhaar et al. reported that utilizing a stimulator of interferon genes (STINGs) agonist promoted effector T-cell infiltration and anti-tumor effects in a CXCR3-dependent manner [125]. Mice were implanted with KPC-derived tumor cells on a single flank, and then treated with a STING agonist, which significantly reduced the tumor burden, increased the CD8^+^/CD4^+^ T-cell ratio, and increased CXCR3^+^ CD8^+^ T cells in the PDAC TME [125]. An analysis of tissue homogenates, using an unbiased multiplex cytokine array, identified CXCL9 and CXCL10 as being significantly elevated, indicating their role in attracting CXCR3^+^ T cells. To ensure that CXCR3 expression was required for CD8^+^ T-cell infiltration and anti-tumor effects, Vonderhaar et al. treated CXCR3 KO mice, implanted with KPC tumors as well, with the same STING treatment regimen and found no anti-tumor effects and no difference in CD8^+^ T-cell infiltration compared to control, while the CCR5 receptor expression was unchanged [125]. This confirmed that anti-tumor effects were dependent on CXCR3 expression by T cells and not CCR5.

Supporting the possible anti-tumor role of CXCR3 and CCR5 and their ligands in PDAC, Gorchs et al. found that an increased CXCR3 ligand expression was associated with an increased number of T cells in tumor-rich areas [126]. Using tissues obtained from patients with resectable pancreatic cancer (n = 19), with central tumor tissue, peripheral tumor tissue, and non-tumor tissue all obtained, the tissues were cultured for 48 h to examine chemokine secretion or stained for various markers of T-cell infiltration [126]. The authors found that CXCR3 ligands (CXCL9, 10, and 11) were all expressed at higher rates by central tumor tissue than non-tumor tissue, while there was no difference in CXCR3 and CCR5 ligand expression across tissues. In examining the tissues via IHC, the authors found that CD8^+^ T cells were significantly fewer in areas closer to the central tumor than in the total stroma, suggesting that cytotoxic T cells are mostly excluded from interacting with malignant epithelial cells in the PDAC TME [126]. To examine whether chemokine secretion was influencing CD8^+^ T-cell localization, the authors examined the T-cell count per mm^2^ of tissue sections and found that tissue with a high stromal chemokine expression had significantly less CD8^+^ T cells than tissues with less chemokine expression. Additionally, high stromal chemokine levels correlated with fewer cytotoxic T cells [126]. However, when examining the relative localization of CD8^+^ T cells based on the chemokine expression pattern, the authors calculated the ratio of CD8^+^ T cells within reach of tumor cells relative to CD8^+^ T cells in the stroma, and found that tissues with a high CD8^+^ tumor cell/stroma ratio had higher levels of chemokines CXCL10, CXCL11, and CCL8 [126]. Thus, this suggested that, while the high, non-specific secretion of chemokines is associated with poor cytotoxic T-cell infiltration, the specific, localized secretion of CXCR3 and CCR5 ligand chemokines near tumor cells correlates with increased CD8^+^ T-cell infiltration. Thus, possibly creating a focused, chemokine gradient at sites of malignant epithelial cells can be utilized as a strategy to increase cytotoxic CD8^+^ T-cell infiltration in PDAC.

#### 4.1.2. NK Cells

Apart from cytotoxic CD8^+^ T cells, NK cells are a key player in anti-tumor immune responses as well. While acting as a complimentary part of the cytotoxic immune response, NK cells function drastically differently from CD8^+^ T cells [127]. NK cells signal through a variety of activating and inhibitory receptors that work to balance functional activity. Whether cytotoxic functions are activated against target cells is determined through the recognition of a “missing self” by NK cells, where MHC-I expression by cells elicits an inhibitory signal to NK cells [127]. While the effect of cytotoxic CD8^+^ T cells has been extensively studied in cancer immunotherapy, the role of NK cells, especially with regard to PDAC immunotherapies, has not been as thoroughly investigated. Recent evidence has suggested though that NK cells are critical to anti-tumor responses elicited by anti-PD1 ICI [128]. Hsu et al. have recently shown, using murine models of breast, prostate, and colorectal tumors, that NK cells are indispensable to the full therapeutic effect of anti-PD1 therapies [128]. This was highlighted when NK-cell-depleted mice treated with anti-PD1 experienced significant increases in tumor growth compared to mice with NK cells treated with anti-PD1 [128]. Thus, understanding NK cell infiltration and the mechanisms by which they accumulate, in relation to chemokines, is essential to improving immunotherapy treatments for PDAC. With regard to PDAC, Lim et al. highlighted that NK cell accumulation in PDAC is minimized due to the lack of CXCR2 receptor expression on NK cells [129]. Analyzing tumor specimens from 80 patients with pancreatic cancer via flow cytometry found very few NK cells among the tumor-infiltrating lymphocytes (TILs) of patients with pancreatic cancer, compared to other lymphocyte subsets such as T cells and B cells [129]. Additionally, an examination of tumor tissue via qPCR analysis found that the tumors expressed high levels of chemokines such as CXCL3 and CXCL5, ligands for the CXCR2 receptor [129]. Upon comparison, via flow cytometry, of peripheral blood NK cells in healthy donors and patients with pancreatic cancer, NK cells from pancreatic cancer patients had a downregulated CXCR2 cell surface expression, suggesting that CXCR2 possibly dictates NK cell localization in tumors [129]. Supporting this notion, NK cells that were present in tumors were positive for CXCR2. This work highlighted how chemokine receptor cell surface expression, in addition to ligand elaboration by tumor cells, is vital for NK cell trafficking and can serve as a potential strategy to improve NK cell trafficking.

Further examining chemokines that influence NK cell trafficking in PDAC tumors, Fitzgerald et al. uncovered that treatment with BXCL701, a pan-dipeptidyl peptidase (DPP) inhibitor, and anti-PD1 induced an influx of NK cells and was associated with anti-tumor responses in a subcutaneous, murine KPC-derived mT3-2D model of PDAC [12]. Additionally, an analysis of the tumor homogenates from treated vs. control mice via a multiplex cytokine panel highlighted intra-tumoral increases in CXCL9 and CXCL10 [12]. Furthermore, an analysis of infiltrated intra-tumoral NK cells identified a significant increase in CXCR3^+^ NK cells in BXCL701+anti-PD1-treated mice compared to control [12]. This highlights how the creation of intra-tumoral chemokine gradients can potentially induce the infiltration of NK cells, helping facilitate an adaptive immune response and leading to an anti-tumor response. Additionally, Chibaya et al. found that the induction of a senescence-associated secretory phenotype (SASP), using small-molecule MEK and CDK4/6 inhibitors, led to immunosuppression through the activation of the enhancer of zeste homolog 2 (EZH2) [130]. Blocking EZH2 in orthotopic, murine, KPC tumors, treated with MEK and CDK4/6 inhibitors, subsequently led to an increase in intra-tumoral CCL2 and CXCL9/10 production and was associated with NK- and T-cell influx and anti-tumor responses [130]. Furthermore, the overexpression of CCL2 by KPC cell lines, which were, then, orthotopically implanted in mice, drove NK-cell, but not T-cell, accumulation in tumors and extended the overall survival of mice [130]. Surprisingly, treatment with an anti-CCL2 antibody of KPC tumors treated with MEK and CDK4/6 inhibitors, along with an EZH2 blockade, led to a significant reduction in anti-tumor effects, highlighting that NK cell accumulation was critical for tumor regression [130]. Thus, this suggests that CCL2 is critical for NK cell accumulation and anti-tumor responses in a murine KPC PDAC model. Overall, these studies demonstrate that the induction of intra-tumoral chemokine production, through either small-molecule inhibitors or epigenetic reprogramming, are potential strategies to induce NK cell accumulation.

### 4.2. Promoting Pro-Tumor Immune Responses

#### 4.2.1. Tumor-Associated Macrophages

Macrophages are unique cells that bridge the innate and adaptive immune response. Not only do they phagocytose and destroy pathogens, macrophages can also present antigens via MHC-I and MHC-II to activate the adaptive immune response. However, their role in tumor suppression vs. promotion is highly dictated by the signal they receive from their surrounding microenvironment, leading to polarization and an M1 or M2 phenotype. M1 macrophages are generally associated with anti-tumor responses, given their induction in response to IFN-γ produced by NK cells and T-helper 1 (Th1) cells [131]. M1 macrophages, in turn, secrete pro-inflammatory cytokines, such as IL-6, IL-12, and TNF-α, further leading to Th1 polarization and anti-tumor effects. Conversely, M2 macrophages work to turn down the inflammatory response, secreting cytokines such as IL-10 and TGF-β, resulting in immunosuppression [131]. Of note, this simplified framework does not truly reflect the complexity of the macrophage polarization in the TME, where macrophage polarization is dynamic and reversible, but is helpful in establishing a baseline for macrophage function. With regard to the PDAC TME, macrophages are derived from a mixed population of tissue-resident cells and circulating monocytes and are more likely to exhibit an M2 phenotype, leading to the promotion of tumor growth [132]. As has already been discussed, key chemokines in recruiting monocytes, leading to the accumulation of TAMs in the PDAC TME, include CCL2, CXCL8, and CXCL14 [39,53,57,118]. TAMs have also been shown to be recruited by an increased intra-tumoral CCL7 expression, through the signaling of CCR2 on the surface of monocytes, in metastatic renal cell carcinoma, suggesting alternate chemokines induce the accumulation of TAMs as well [133]. TAMs are also a primary source of chemokine production upon accumulating in the PDAC TME. As discussed, TAMs create positive feedback loops, increasing the secretion of chemokines that led to their initial accumulation, such as CCL2, and increase M2 polarization, such as CCL18. In addition, TAMs have been shown to elaborate chemokines such as CCL20, which is known to promote intra-tumoral Treg accumulation and has been shown to be upregulated in pancreatic cancer by qPCR (relative to normal tissue) [134,135]. Thus, TAMs are fundamental to the PDAC TME chemokine network and play a multifaceted role in fostering an immunosuppressive environment, both directly and indirectly.

#### 4.2.2. Tumor-Associated Neutrophils

Neutrophils are the most abundant type of leukocyte in the immune system, representing about 70% of all circulating immune cells at any given timepoint. Neutrophils’ primarily responsibility is to phagocytose foreign pathogens and they normally respond within minutes during the acute phase of inflammation [136]. Some studies have proposed classifying neutrophils similarly to macrophages, with an N1 anti-tumor, pro-inflammatory subset and an N2 pro-tumor, immunosuppressive subset [137]. However, again, similar to macrophages, this classification system appears overly simplified with mixed phenotypes being identified in some tumors, such as primary sarcomas [137,138]. Interestingly, recent studies suggest that most MDSCs, which are a group of heterogenous myeloid cells that can suppress immune response through the induction of CD8^+^ T-cell tolerance and the inhibition of NK-cell cytotoxicity by blocking Stat5 activation, are, in fact, mostly composed of a subset of neutrophils [139,140]. Work by Fridlender et al., using a transcriptomic approach to analyze surface marker expression by neutrophils present in tumors of AB12 mesothelioma tumor-bearing mice, found that naïve neutrophil- and granulocytic-derived MDSCs (g-MDSCs) significantly overlap in terms of mRNA profiles [141]. Interestingly, g-MDSCs and TANs overlapped in terms of immune-related genes upregulated, especially with regard to the antigen presentation and inflammatory cytokine profile [141].

With regard to tumors, studies have shown that neutrophils make up a substantial proportion of immune cell infiltrate, including in PDAC [142]. In the context of PDAC, increased proportions of neutrophils have been associated with a poor prognosis and immunosuppression [143]. While the recruitment of neutrophils to the PDAC TME by chemokines such as CXCL1, CXCL2, CXCL5, and CXCL8 has already been discussed, these chemokines have also been shown to play differing roles, in terms of importance, in attracting differing neutrophil populations. Sano et al. demonstrated that, with regard to the knockout of CXCR2, a receptor for CXCL1, 2, 5, and 8, in LSL-Kras^G12D/+^; Tgfbr2^flox/flox^, and Ptf1a-Cre (PKF) mice, which spontaneously develop PDAC with dense desmoplasia, the infiltration of myeloperoxidase (MPO)-positive neutrophils is significantly decreased compared to control animals [144]. Given that MPO dysregulation has been associated with an increased risk of pancreatic cancer, this suggests that the recruitment of such neutrophil populations promotes tumorigenesis [145]. Similarly, Steele et al. demonstrated, using a KPC mouse model, that the secretion of CXCL1, 2, and 5 from tumor cells increased the MPO-positive neutrophils compared to the pancreas of control mice [92]. With regard to CXCL5 in particular, Nywening et al. further interrogated its role in human PDAC tumors and found a positive correlation between CXCL5 expression and CD15 and neutrophil-elastase-expressing neutrophils [146]. Additionally, neutrophil elastase has been shown to reduce e-cadherin and keratin expression in pancreatic cancer cells, suggesting a possible role in promoting metastasis with increased expression [147]. Further supporting a critical role for CXCL5 in neutrophil accumulation, Chao et al. found, using a KPC mouse model, that KRAS/MEK inhibition led to NF-ĸB activation and the induction of CXCL5 secretion, leading to elevated levels of CD11b^+^Ly6G^+^ and tumor progression [69]. Given these findings, it appears that CXCL chemokines, especially CXCL5, are essential to the accumulation of TANs in the PDAC TME, which work to promote immunosuppression.

#### 4.2.3. T Regulatory Cells

T regulatory cells, like most parts of the immune system, can be either beneficial or harmful depending on the context in which they are found. Defined as CD4^+^CD25^+^FOXP3^+^ T cells, Tregs act to suppress immune responses, through the secretion of inhibitory cytokines (IL-10 or TGF-β), consumption of IL-2, and expression of immune checkpoints to downregulate the antigen presentation and adaptive immune activation [148]. Normally, Tregs function to prevent auto-immunity, suppressing the immune response to ensure homeostasis. However, their presence in the TME can aid and enhance tumor growth. With regard to PDAC, Tregs have been demonstrated to accumulate in the PDAC TME of both mice and humans [149,150,151]. Critically, an increased Treg frequency correlates with tumor metastasis and a poor prognosis in human PDAC patients [152]. In PDAC, multiple chemokines are known to be involved in facilitating Treg recruitment. As previously mentioned, CCL5 production by pancreatic cancer cells appears to facilitate Treg recruitment in PDAC [60]. Supporting this, Tan et al. have also demonstrated, in a Pan02 murine PDAC model, that Tregs preferentially express CCR5 among T cells and disrupting CCL5/CCR5 signaling, either through inhibiting production or inhibiting ligand/receptor binding, significantly reduced Treg infiltration [153]. Additionally, Ene-Obong et al. identified CXCL12, produced by CAFs in the PDAC stroma, as interacting with CXCR4 on the surface of CD4^+^ and CD8^+^ T cells, enhancing their trafficking to the PDAC TME [9]. Using a tissue microarray with PDAC samples from multiple patients (n = 6), the authors found that T cells were significantly higher in pan stromal regions (where activated CAFs were present) than in juxtatumoral regions. While this can lead to the sequestration of CD8^+^ T cells away from the tumor, as the authors pointed out, increasing the trafficking of CD4^+^ cells, especially naïve ones, can lead to the increased accumulation of Tregs due to the immunosuppressive molecular signals present in the PDAC TME [9,154].

In addition to CCL5 and CXCL12, ligands of CCR4 have also been implicated in regulating Treg trafficking in the PDAC TME. Given that CCR4 is expressed on >90% of Treg cells, as shown by Gobert et al. through an FACS analysis of CCR4 expression on peripheral T cells of healthy patients and breast cancer patients, Marshall et al. examined the impact of CCL17 and CCL22 on Treg accumulation in the PDAC TME [155,156]. Using a Pan02 subcutaneous, murine PDAC model, the authors found that the tumors expressed high levels of CCL17 and CCL22, via qPCR analysis, and FOXP3^+^ cells were present [155]. Underscoring the effects of CCL17 and CCL22, the inhibition of CCR4, using a small-molecule inhibitor, significantly decreased the percentage of Tregs in murine tumors compared to control [155]. When combined with anti-cytotoxic T-lymphocyte antigen-4 (CTLA4) ICI therapy, this further decreased the accumulation of Tregs [155]. Further supporting the role of CCL22 in Treg recruitment, Wiedemann et al. identified CCL22 as being produced by intra-tumoral dendritic cells based on an IHC analysis of PDAC tissue specimens [157]. To ensure tumor cells were not the source of CCL22, the co-culture of PBMCs with PaTu8988t pancreatic cancer cells induced the secretion of CCL22 by PBMCs with no CCL22 detected in the supernatant of cancer cells alone [157]. Interestingly, the authors identified that the CCL22 released from PBMCs was due to the IL-1α released from tumor cells. Further supporting the role of IL-1α, the supernatant from PaTu8988t cancer cells + PBMCs that anakinra (a IL-1-receptor-blocking antibody) was added to had a reduced capacity to induce the migration of Treg cells in a trans-well migration assay system, compared to a supernatant without anakinra [157]. As expected, CCL22 levels were significantly reduced in this supernatant as well. Unsurprisingly, this highlights how tumor cells can modify infiltrating immune cells in order to secrete chemoattractants that cause further immunosuppression and promote tumorigenesis.

### 4.3. Pleotropic Chemokines

While multiple chemokines have been discussed in the previous sections, as either promoting or suppressing anti-tumor responses through their effects on immune cell trafficking and function, studies have shown that many of these chemokines may also have alternate and possibly contradictory effects.

While CXCL9 and CXCL10 were discussed as drivers of CD8^+^ T-cell and NK-cell infiltration, other studies have highlighted possible adverse roles of CXCR3 ligands in the context of PDAC. Huang et al. demonstrated, through a CIBERSORT analysis of 182 PAAD samples obtained from The Cancer Genome Atlas (TCGA), that CXCL10 mRNA expression levels positively correlated with tumor cell differentiation and negatively correlated with prognosis [158]. However, the authors also found that CXCL10 mRNA expression negatively correlated with Treg expression and positively correlated with M1 macrophage expression markers [158]. With regard to T-cell exhaustion, Cannon et al. demonstrated, through an analysis of T-cell-related genes and CXCL9, 10, and 11 expression in PAAD samples from the TCGA dataset, that T-cell exhaustion, and the PD-1 and PD-L1 pathways correlated with CXCL9, 10, and 11 expression [159]. These findings were further supported by analyzing the PDAC microarray data (n = 123 samples) and ex vivo treatment of murine splenocytes with CXCL10, which induced the mRNA expression of immunosuppressive markers such as LAG3 and CTLA4 [159]. Studies have also suggested that CXCR3^+^ regulatory T cells can be attracted to the PDAC TME, a plausible scenario given that 30–40% of peripheral Tregs have been found to express CXCR3 [160,161]. However, a more thorough investigation of CXCR3^+^ Tregs in PDAC is needed to fully understand the role of CXCL9/10/11 in Treg trafficking.

CCL5 has been found to have a more nuanced role in immune cell trafficking in PDAC than previously believed. As already discussed, CCL5 production by pancreatic cancer cells appears to increase Treg accumulation, which also express CCR5 at higher rates than effector T cells [60,63]. However, work by Huffman et al. and Romero et al., highlighting its role in promoting anti-tumor CD4^+^ responses in response to CD40 agonists and an inflammatory, effector T-cell phenotype in PDAC, respectively, demonstrates how CCL5’s role is possibly context-dependent [34,123]. One possible explanation for this discrepancy is that cancer cells may upregulate CCL5 in order to foster initial immunosuppression, but, upon immune activation (such as by therapies like CD40 agonists), immune cells, such as monocytes and dendritic cells, upregulate the expression in order to foster immune activation and adaptive immune response.

CXCL12, almost universally considered as a negative factor in the PDAC TME, may also have a more diverse role than once considered. While known to attract CD8^+^ T cells, acting to sequester and misdirect them away from tumoral cells and towards CAFs, recent work suggests that CXCL12 may also be necessary for T cells to eliminate cancer cells [162]. Lin et al., using spheroid models with PDAC cancer cells derived from KPC tumor-bearing mice, found that cancer cells resistant to a T-cell attack in vitro (which were tumor-educated T cells obtained from draining lymph nodes of tumor-bearing mice) universally had a decreased expression of CXCL12 [162]. Adding back CXCL12 to these tumor spheroid models restored T-cell functionality and reversed the resistant phenotype [162]. Thus, this study highlights that conventional thinking about CXCL12, where it has been implicated as promoting immune evasion, appears incomplete, and has yet to be fully understood.

CCL2 has also been shown to positively impact tumorigenesis in PDAC, through its ability to attract TAMs, improve cancer cell self-renewal capabilities, and enhance cancer cell survival in response to radiotherapy [33,53,58]. However, the conventional wisdom that CCL2 only promotes immunosuppression is also being challenged by recent studies, highlighting potential beneficial effects in PDAC. Long et al. demonstrated that CD40 agonism induced IFN-γ and CCL2 release, which work in tandem to polarize and attract monocytes to infiltrate PDAC tumors and deplete fibrosis [163]. Using a KPC mouse PDAC model, the authors highlighted how CD40 agonist therapy induced a Ly6C^+^CCR2^+^ subset of monocytes to accumulate in the PDAC TME, which was dependent on CCL2 secretion by inflammatory and resident macrophages [163]. When combined with IFN-γ signaling, induced by CD40 agonism as well, these monocytes possessed anti-fibrotic activity and could be redirected to regulate the MMP expression profile in PDAC tumors and contribute to the degradation of fibrosis [163]. This degradation of fibrosis could then be applied to enhance the efficacy of gemcitabine chemotherapy [163]. In addition to enhancing the trafficking of anti-fibrotic monocytes, CCL2 has also recently been demonstrated as being critical to orchestrating NK-cell-mediated anti-tumor effects in PDAC models. As previously discussed, Chibaya et al. found that blocking EZH2 in orthotopic, murine, KPC tumors, treated with MEK and CDK4/6 inhibitors, subsequently led to an increase in intra-tumoral CCL2 and an influx of NK cells [130]. The overexpression of CCL2 in PDAC cell lines subsequently increased the NK cell accumulation and enhanced mouse survival, contrary to current wisdom which would suggest overexpressing CCL2 would increase TAM accumulation and worsen outcomes [130]. Thus, this suggests that CCL2 is critical for NK cell accumulation and plays a more multifaceted role in the anti-tumor response than previously believed. As with many of these chemokines, their biology with regard to PDAC will have to be more thoroughly investigated, especially in relation to their spatial localization in the PDAC TME. It appears highly likely that given chemokines’ innate roles, functioning to create gradients which attract immune cells, their source and localization in the PDAC TME determines the degree to which anti-tumor responses are generated.

## 5. Chemokines and Patient Outcomes in PDAC

Since chemokines are essential to not only facilitating the immune response, but also influencing tumor progression, considerable research has focused on the effects of chemokines in relation to patient outcomes in PDAC. Most work has focused on examining chemokine expression intra-tumorally and, in the peripheral circulation, quantifying both mRNA and protein expression. The utility of identifying the effect of increased/decreased chemokine signaling and/or receptor expression in the PDAC TME lies in the fact that these results, if correlated with patient outcomes, can yield insights into which chemokines to attempt to possibly target therapeutically. While caution has to be used in interpreting correlative studies, given that the results are not indicative of causation, they can provide a starting point for better understanding chemokine biology in PDAC.

### 5.1. CC Chemokines

CCL2, one of the most studied chemokines, was investigated by Sanford et al. in relation to CD8^+^ T-cell infiltration and patient survival. The authors noted, studying resected PDAC specimens from patients that had undergone pancreaticoduodenectomy (n = 483) for the mRNA expression of CCL2, that patients with high CCL2/low CD8^+^ T-cell signatures had significantly decreased overall survival as compared to patients with the inverse signature (*p* < 0.0002) [164]. CCL18, another chemokine discussed in terms of promoting PDAC progression and immunosuppression, is also reported to negatively impact overall survival. Meng et al., through an IHC analysis of the CCL18 expression in samples from 62 patients that underwent PDAC resection, found that patients with CCL18 expression in either cancer cells or mesenchymal cells had significantly shorter overall survival as opposed to patients without expression in either cell type [85]. Surprisingly, no statistically significant association between overall survival and CCL18 expression was found in patients with CCL18 expression in only cancer cells or mesenchymal cells [85]. Additionally, the increased expression of CCL20 has also been correlated with decreased overall survival in PDAC patients [165].

### 5.2. CXC Chemokines

Regarding CXC chemokines, there also exist considerable discrepancies in whether individual chemokines are beneficial or harmful to prognosis. Utilizing a multiomics and bioinformatics approach, Jing et al. analyzed the mRNA expression in PDAC of CXC chemokines using the ONCOMINE and Gene Expression Profiling Analysis (GEPIA) datasets. Prognostic significance was then evaluated through Kaplan–Meier curves, by categorizing samples as high- or low-expressing for each particular chemokine. The authors found that PDAC patients that had tumors with a high expression of CXCL5, CXCL9, CXCL10, or CXCL17 had significantly worse overall survival (*p* < 0.05) [166]. CXCL17, while not previously discussed, has actually been found to induce the accumulation of immature dendritic cells and promote anti-tumor responses in intraepithelial precursor lesions of PDAC [167]. Thus, it is surprising to see that its expression is a negative prognostic factor [167]. Further analysis by Zhang et al. of CXCL5, based on western blot data and tissue microarray staining of PDAC patient samples, found that high CXCL5 protein expression was correlated with a poor prognosis (*p* = 0.001) [168]. Additionally, Fang et al. identified increased intra-tumoral levels of CXCL8 as correlating with worse overall survival in PDAC patients [169]. Interestingly, the authors also highlighted that increased intra-tumoral levels of CXCL8 correlated with increased serum levels of CXCL8, suggesting that serum IL-8 levels can possibly be utilized as a factor in determining the prognosis of PDAC patients [169].

CXCL9 and 10, both ligands for CXCR3, have been previously discussed regarding their pleotropic nature in both promoting and suppressing immune responses. In addition to Jing et al., others have shown that CXCL10 expression is correlated with poor survival [158,160]. While Huang et al. relied on publicly available datasets, similar to Jing et al., Lunardi et al. analyzed tissue samples from 48 patients with resectable PDAC and found that a high CXCL10 expression correlated with decreased median overall survival [158,160]. However, reports have also found that both ligands can be correlated with increased survival in PDAC patients. Analyzing plasma levels of CXCL9 and 10 in 200 patients receiving palliative chemotherapy for PDAC and survival time, Qian et al. found that higher circulating CXCL9 and 10 levels were correlated with significantly longer overall survival in advanced PDAC patients [170]. Interestingly, Cannon et al., analyzing CXCL9 and 10 expression bioinformatically and CXCR3 expression via the IHC staining of patient PDAC samples, found that a higher ligand expression was associated with shorter overall survival, while an increased CXCR3 expression was associated with better survival [159]. This observation can possibly be accounted for in part by where the ligands have multiple effects, both anti-tumor and immunosuppressive, but CXCR3, given its predominant expression on effector T cells and NK cells, accumulation leads to greater anti-tumor activation and patient survival [159,171].

CXCL12, with its roles in tumor cell dissemination and immune attack evasion, unsurprisingly has also been associated with a poor prognosis. Using biopsy samples from 76 patients with PDAC that had surgical intervention to attempt and remove tumors, D’Alterio et al. used IHC to analyze samples for CXCL12 expression. When comparing patients, based on a high or low CXCL12 expression, to survival data, the authors found that a high CXCL12 expression was a prognostic indicator for worse recurrence-free survival (RFS) and cancer-specific survival (CSS) [172]. Interestingly, the high expression of known receptors for CXCL12, CXCR4, and CXCR7 in biopsy samples was indicative of worse RFS or CSS, respectively, but not both [172]. To confirm CXCL12’s prognostic role, the authors evaluated the expression in an independent cohort of fine-needle aspiration cytology biopsies (n = 20). CXCL12 was detected in 14/20 samples and, when sorted according to low or high CXCL12 expression, based on an IHC analysis, patients with a high expression showed a median survival of 3 months, while patients with a low expression had a median survival of 12 months (*p* = 0.02) [172].

### 5.3. CX3C Chemokines

CX3CL1, the only chemokine of the CX3C family, has been shown to be chemotactic for CD8^+^ T cells, NK cells, monocytes, and dendritic cells [173]. Recently, its expression has also been investigated as a possible prognostic factor in PDAC. Xu et al. demonstrated, through an IHC analysis of 105 PDAC specimens, that CX3CL1 expression was detected in 77.1% of all samples and CX3CR1 in 66.7% [174]. When analyzed via a multivariate analysis, the authors identified high CX3CL1 expression as one of the independent negative prognostic factors with regard to overall survival [174]. Furthermore, patients expressing both high levels of CX3CL1 and CX3CR1 had significantly worse overall survival compared to patients with low/no detectable expression (*p* = 0.009) [174]. Celesti et al. further examined CX3CL1/R1 expression early on in PDAC development, analyzing the expression of both the ligand and receptor in 104 human PDAC and PanIN biopsy samples in patients that underwent resection for PDAC. While >50% of samples expressed either CX3CL1 or CX3CR1, surprisingly, survival was found to be significantly improved in patients with CX3CR1-expressing tumors [46]. No difference in survival was seen in patients with tumors expressing or not expressing CX3CL1 [46]. The authors discuss this discrepancy, in relation to the results of Xu et al., and suggest that their results of improved overall survival with CX3CR1 expression may be reflective of the samples they analyzed being from patients with earlier disease. They support this suggestion by highlighting the fact that tumor grade was also a predictor of patient survival in their tumor analysis, with CX3CR1 expression retaining prognostic value when adjusted for tumor grade as well [46]. Thus, these studies suggest that an increased CX3CL1/R1 expression is prognostic for worse survival in PDAC patients.

## 6. Chemokines and Immunotherapy Approaches

Cancer immunotherapy has been underwhelming in PDAC [7]. Clinical trials utilizing immunotherapies, such as ICIs, have consistently shown minimal to no improvement [175]. Given that the infiltration of cytotoxic immune cells, such as CD8^+^ T cells and NK cells, has been correlated with increased immunotherapy efficacy in PDAC, approaches combining treatments with modalities that increase chemokine elaboration, to attract immune cells, are an attractive approach [7,8].

### 6.1. Checkpoint Inhibitors

ICIs, targeting the PD1-PD-L1 axis and CTLA4, have been thoroughly investigated in PDAC and found to perform poorly in most clinical trials [176]. Currently, the success of such approaches is limited only to a small subset of PDAC patients (1–2%) with microsatellite instability (MSI) high tumors. In order to improve the ICI anti-tumor effects, researchers have combined these treatments with approaches to increase chemokine elaboration, hoping to promote tumor-infiltrating lymphocyte (TIL) infiltration. As previously discussed, Fitzgerald et al. found that the inhibition of DPPs in murine PDAC models led to increases in the intra-tumoral CXCL9/10 levels [12]. When combined with anti-PD1 treatment, this induced an anti-tumor response and induced immune memory in treated animals, which were resistant to tumor growth when rechallenged with tumors after the initial treatment [12]. With regard to immunochemotherapy, Ho et al. combined gemcitabine, currently the first-line chemotherapy treatment for PDAC patients, with anti-PD1 therapy to see if this enhanced the anti-tumor effects against pancreatic cancer liver mets in a murine Pan02 cancer model [177]. The authors found that the increased infiltration of CD4^+^ Th1 cells and M1-polarized macrophages in liver mets was associated with increases in CCL2 and CXCL10, compared to anti-PD1 therapy alone [177]. Additionally, Jing et al., using a murine STING agonist in two, orthotopic KPC-derived murine PDAC tumor models, found that STING agonism promoted the reprogramming of chemokine production by macrophages, dendritic cells, and pancreatic cancer cells [178]. Specifically, the authors found that multiple chemokines, including CCL2, CCL3, CCL4, CCL5, CXCL1, CXCL2, CXCL9, and CXCL10, were elevated in mice treated with the STING agonist, and this was associated with prolonged survival in mice [178]. Given these promising results, STING agonists, combined with anti-PD1 and/or anti-CTLA4 therapy, have entered clinical trials in multiple ICI refractory solid tumors, including PDAC, to identify if enhancing chemokine secretion intra-tumorally will result in an increased anti-tumor response [179].

### 6.2. Cellular Therapies

Cellular therapies, such as chimeric antigen receptor T-cells (CAR-T), have become promising tools in targeting tumors via specific antigens. While highly effective in CD19-expressing hematological malignancies, such as diffuse large B-cell lymphoma (DLBCL), there has been less success in solid tumors with CAR-T [180]. One of the reasons for this is the inability of CAR-T cells to sufficiently infiltrate tumors while also avoiding exhaustion. One approach to attempt to overcome this has been to engineer CAR-T cells that express chemokines upon antigen stimulation. IL-7- and CCL19-expressing CAR-T cells (7 × 19 CAR-T cells) have demonstrated considerable promise in murine PDAC models, with initial studies also suggesting efficacy in humans [181,182]. Adachi et al. first demonstrated that CAR-T cells, specific for endogenous murine mesothelin, engineered to express IL-7 and CCL19 upon CAR stimulation, demonstrated significant anti-tumor effects in a subcutaneous, murine Pan02 PDAC model [181]. IL-7 was selected, given its role in enhancing T-cell proliferation and survival, while CCL19 is a T-cell chemoattractant, signaling via CCR7 [183,184]. While the injected CAR-T cells showed a significant effect, which was dependent on IL-7 and CCL19 secretion, the depletion of host T-cells abrogated the anti-tumor effects, suggesting co-operation between adoptively transferred and native T-cells in facilitating tumor rejection [181]. A similar approach was utilized by Pang et al. to study the effects of anti-human mesothelin 7 × 19 CAR-T cells where AsPC-1 human pancreatic cancer cells were subcutaneously inoculated in mice. Significant anti-tumor effects were observed, where 7 × 19 mesothelin-targeting CAR-T proved more efficacious than CAR-T only targeting mesothelin. Based on these results, a Phase I trial was initiated where the treatment of one patient with advanced pancreatic cancer with anti-mesothelin resulted in almost complete tumor disappearance 240 days post IV infusion of 7 × 19 CAR-T cells [182]. These promising early results suggest that the addition of inducible chemokine expression can potentially optimize CAR-T therapies for the treatment of PDAC.

In addition to optimizing chemokine expression, others have tried to increase immune infiltration through the upregulation of chemokine receptors on immune cells. Given that PDAC is known to express increased amounts of CXCL8, Jin et al. modified anti-CD70 CAR-T cells to express CXCR1 and CXCR2 (the receptors for CXCL8) [185]. PANC-1 cells were implanted subcutaneously in a mouse model, and then treated with two doses of fractionated local radiation (4.5 Gy/dose), which enhanced CXCL8 secretion by tumors. Mice were then treated with CAR-T cells overexpressing CXCR1 or CXCR2, which led to significant tumor regression and appeared to demonstrate a synergy between radiotherapy and chemokine-targeted CAR-T treatment [185]. Building upon this approach, Whilding et al. highlighted that CAR-T cells targeting ⍺Vβ6, an integrin highly expressed by multiple solid tumors, and overexpressing CXCR1 or CXCR2 demonstrated increased chemotaxis towards IL-8-expressing tumors and conditioned media that contained the chemokine [186]. CAR-T cells expressing CXCR2 appeared more efficacious at tumor homing than CXCR1-expressing CAR-T cells, while also decreasing the tumor burden and increasing T-cell infiltration relative to CAR-T cells not expressing CXCR2 [186]. Apart from CXCR1/2, Lesch et al. have also demonstrated that overexpressing CXCR6 on CAR-T cells can enhance trafficking and anti-tumor effects [187]. In both subcutaneous, murine PDAC models, with CAR-T cells targeting the tumor-associated antigen epithelial cell adhesion molecule, or orthotopic, murine PDAC models, with CAR-T cells targeting mesothelin, T cells exhibited enhanced accumulation, exerted sustained anti-tumoral activity, and prolonged animal survival only when expressing CXCR6 [187].

In addition to CAR-T cells, CAR-NK cells have also emerged as a promising cellular therapy option for patients [188]. However, they have also been plagued by similar issues to CAR-T cells, especially with regard to intra-tumoral accumulation. Overexpressing the chemokine receptor, CXCR2, on NK cells has been shown to increase migration to renal cell carcinoma tumors expressing cognate ligands, such as CXCL8 [189]. Additionally, Müller et al. demonstrated that anti-EGFRvIII CAR-NK cells, engineered to express CXCR4, demonstrated increased chemotaxis towards CXCL12/SDF1α-secreting glioblastoma cells, leading to increased survival and tumor regression in a mouse model of glioblastoma [190]. While not yet tested in PDAC models, given that PDAC has been shown to express both CXCL8 and CXCL12, NK/CAR-NK cells overexpressing CXCR2 and/or CXCR4 could prove to be a powerful cellular therapy option in PDAC as well.

### 6.3. Vaccines

Cancer-specific vaccines have been investigated for decades in numerous cancer types [191]. Recent work in PDAC has showcased limited, yet encouraging, outcomes that suggest cancer vaccines can become an additional treatment modality in PDAC [192]. One approach has utilized the GVAX vaccine, which is composed of irradiated pancreatic cancer cells that have been engineered to secrete granulocyte-macrophage colony-stimulating factor (GM-CSF) in order to stimulate immune responses [193]. Interestingly, when GVAX was recently combined with PEGPH20, a stromal hyaluron (HA)-degrading agent, in a metastatic, murine PDAC model, an increase in effector memory T cells (which were CCR7^−^), along with increased tumor-specific IFN-γ, was observed [194]. Significantly, murine tumors upregulated CXCR4 upon GVAX treatment, which was mitigated with a combination treatment with PEGPH20 [194]. Additionally, these mice exhibited increased survival compared to control and vaccine-alone mice. When examining human PDAC tumors, from patients that were treated with GVAX, decreased stromal CXCR4 expression correlated with decreased stromal HA and increased expression of cytotoxic markers [194]. Thus, the efficacy of immune-stimulatory vaccines such as GVAX may depend on the targeted inhibition of certain chemokine pathways, such as CXCL12/CXCR4. Overall, more research is required to fully delineate the effects of cancer vaccines on PDAC chemokine elaboration.

### 6.4. Oncolytic Viruses

Oncolytic viruses have emerged as an innovative and promising field of immunotherapy. While the first oncolytic virus was approved for the treatment of nasopharyngeal cancer in 2006 in China, the field has made minimal progress since, especially with regard to PDAC [195]. However, recent work has attempted to combine virotherapy with modern immunotherapy treatments, such as anti-PD1, to see if this can increase the anti-tumor effects. Veinalde et al. demonstrated that combining virotherapy using MV-NIS, an oncolytic agent currently in clinical trials, with anti-PD1 significantly prolonged survival in a murine, KPC-derived PDAC mouse model [196]. An immune pathway analysis of tumors treated with combination therapy highlighted enhanced adaptive immunity, and cytokine and chemokine signaling [196]. Similarly, a Phase Ib trial of pelareorep, an oncolytic reovirus, combined with anti-PD1 and chemotherapy, showed encouraging efficacy in PDAC patients that had progressed after first-line treatment, with disease control achieved in 3 out of 10 patients [197]. Interestingly, treatment with this combination induced changes in peripheral blood chemokines, with significant increases in CXCL9, CXCL10, and CXCL11 after the second treatment cycle [197]. Additionally, there was no significant increase in the Treg-attractive chemokines, CCL22 and CXCL12. While more investigation is needed in further trials, it is possible that such viruses can be used to increase the inflammatory nature of the PDAC TME through the induction of chemokine expression.

## 7. Targeting Chemokine Receptors and Ligands

Given all the pro-tumor effects that have been attributed to chemokine signaling in the PDAC TME, work has focused over the last decade on blocking chemokine ligand/receptor interactions. These efforts are documented by the specific axis being targeted. While numerous chemokines have been targeted preclinically in animal models, clinical work has focused on the most extensively studied ligand/receptor pairs. Often, these trials have been combined with either chemo- or immunotherapy treatments. Table 1 summarizes active and recently completed clinical trials that target chemokine receptors and ligands in the treatment of PDAC.

### 7.1. CCR2/CCL2

Given the multiple reports that have classified CCL2 expression as having a role in the accumulation of CCR2^+^ TAMs in the PDAC TME, which has been associated with a decreased prognosis, trials have been initiated to understand if blocking CCR2 enhances anti-tumor effects in PDAC. Recently, a potent CCR2 antagonist, PF-04136309, has been studied in combination with chemotherapy in pancreatic cancer patients with borderline and resectable PDAC (BRPC) [198]. When combined with FOLFIRINOX (fluorouracil [5-FU], leucovorin, irinotecan, and oxaliplatin), treatment was well-tolerated and resulted in partial responses in patients that were not seen in patients treated with chemotherapy alone [198]. Treatment also resulted in a reduction in TAMs and increased intra-tumoral T-cell infiltration [198]. However, another Phase Ib study with PF-04136309, where it was combined with nab-paclitaxel/gemcitabine in the first-line setting for metastatic PDAC, found no increased efficacy signals relative to nab-paclitaxel/gemcitabine alone, and raised safety concerns for pulmonary toxicity occurring in 24% of study participants [199]. Currently, no active studies are being conducted using this agent in PDAC. Another CCR2 antagonist, CCX872-B, has also been investigated in metastatic PDAC. In a Phase Ib trial, the combination of CCX872-B and FOLFIRINOX resulted in better overall survival in patients with the combination compared to published data for FOLFIRINOX monotherapy (29% vs. 19% at 18 months) [200].

Additionally, a Phase I trial of a human IgG1 monoclonal antibody, carlumab, that is specific for CCL2 in patients with solid tumors refractory to standard treatments demonstrated no objective anti-tumor response out of 44 patients (with 1 PDAC patient among the cohort) [201]. Since, no further trials targeting CCL2 have been conducted.

### 7.2. CCR4

CCR4 has recently emerged as a target, given its association with increased Treg trafficking in PDAC [155]. Mogamulizumab, an anti-CCR4 human monoclonal antibody, has been tested in combination with anti-PD1 therapy in patients with advanced or metastatic solid tumors [202]. Among 15 patients who were enrolled with pancreatic adenocarcinoma, there was one confirmed and two unconfirmed responses, with no dose-limited toxicities observed [202]. Additionally, populations of Tregs (CD4^+^CD45RA^−^FOXP3^+^) decreased while CD8^+^ T cells increased among tumor-infiltrating lymphocytes [202]. While encouraging, a subsequent Phase I trial involving mogamulizumab, combined with either anti-PD1 or anti-CTLA4 therapy, in patients with pancreatic cancer demonstrated no responses among 24 patients in an expansion cohort [203]. While a decrease in peripheral blood and intra-tumoral Tregs was also seen, this study demonstrates that a CCR4 blockade alone is not sufficient to enhance the anti-tumor effects. An additional study, investigating mogamulizumab combined with nivolumab in locally advanced or metastatic solid tumors, demonstrated a similar lack of efficacy in pancreatic cancer (0 objective response in 18 patients) [204].

### 7.3. CCR5

CCR5 antagonism is already an approved treatment modality. Maraviroc is a Food and Drug Administration (FDA)-approved anti-retroviral therapy for the treatment of HIV, due to the fact that HIV utilizes CCR5 as a receptor to infect cells [205]. Given that reports have demonstrated that CCR5 can be utilized by Tregs to increase accumulation in the PDAC TME, clinical trials have been initiated using CCR5 antagonists, in combination with chemo- or immunotherapy, in PDAC patients. BMS-813160 is a dual antagonist of both CCR2 and CCR5 currently being tested in two Phase I/II trials for PDAC, one combined with chemotherapy or anti-PD1 and the other combined with anti-PD1, gemcitabine, and nab-paclitaxel (Table 1). Both trials have not yet reported results. Another trial, NCT03767582, is currently testing BMS-813160 with anti-PD1 therapy, with or without GVAX vaccine treatment, in locally advanced PDAC (LAPC). Preliminary results have identified optimum doses and found that the combination is safe and tolerated by patients [206]. The trial is proceeding to the Phase II portion.

**Table 1:** Clinical trials targeting chemokine pathways in PDAC and outcomes. Multiple different chemokine pathways have been/are being targeted, with some demonstrating modest clinical benefits and anti-tumor effects.

Pathway Targeted	Clinical Trial Number	Study/Patient Info.	Chemokine-Targeted Therapy	Additional Treatment	Outcome	Reference
**CCR2/CCL2**	NCT01413022	Phase Ib, borderline resectable pancreatic cancer, n = 33	CCR2 antagonist (PF-04136309)	FOLFIRINOX	Well-tolerated, 49% objective tumor response	[198]
	NCT02732938	Phase Ib/II, metastatic PDAC, n = 21	CCR2 antagonist (PF-04136309)	Gemcitabine Nab-paclitaxel	No additional benefit, high incidence of pulmonary toxicity (24%)	[199]
	NCT02345408	Phase Ib, locally advanced or metastatic, non-resectable PDAC, n = 50	CCR2 antagonist (CCX872-B)	FOLFIRINOX	Well-tolerated, overall survival of 29%	[200]
	NCT03778879	Phase I/II, PDAC	CCR2 antagonist (CCX872-B)	Stereotactic body radiation therapy (25 Gy in 5 fractions)	Withdrawn due to lack of CCX872-B availability	CT.gov
	NCT03851237	Phase I, PDAC	CCR2 imaging/therapeutic agent (64Cu-DOTA-ECLIi)	Correlating CCR2 expression with response to standard of care chemotherapy/CCR2-targeted therapy	Patient recruitment underway	CT.gov
	-	Phase I, advanced solid tumors, n = 44 (1 PDAC)	Anti-CCL2 antibody (Carlumab, CNTO 888)	-	Well-tolerated, 0% objective anti-tumor response	[201]
**CCR4**	NCT02476123	Phase I, advanced solid tumors, n = 96 (15 pancreatic adenocarcinoma)	Anti-CCR4 antibody (Mogamulizumab)	Nivolumab (anti-PD1)	Well-tolerated, 1 confirmed objective response in PDAC	[202]
	NCT02301130	Phase I, advanced solid tumors, n = 64 (27 pancreatic cancer)	Anti-CCR4 antibody (Mogamulizumab)	Durvalumab (anti-PD1) or Tremelimumab (anti-CTLA4)	Well-tolerated, 0% ORR in pancreatic cancer	[203]
	NCT02705105	Phase I/II, locally advanced or metastatic solid tumors, n = 114 (18 pancreatic cancer)	Anti-CCR4 antibody (Mogamulizumab)	Nivolumab	Acceptable tolerability, no enhanced anti-tumor effects, 0% ORR in pancreatic cancer	[204]
**CCR5**	NCT03496662	Phase I/II, PDAC, n = 40	CCR2/CCR5 dual antagonist (BMS-813160)	Nivolumab Gemcitabine Nab-paclitaxel	Results not yet reported	CT.gov
	NCT03184870	Phase I/II, colorectal cancer and PDAC, n = 332	CCR2/CCR5 dual antagonist (BMS-813160)	Nivolumab or FOLFIRI or Gemcitabine + Nab-paclitaxel	Results not yet reported	CT.gov
	NCT03767582	Phase I/II, PDAC	CCR2/CCR5 dual antagonist (BMS-813160)	Nivolumab with or without GVAX	Safe dosage identified, proceeding to Phase II portion	[206]
	NCT05940844	Phase I, treatment-refractory metastatic solid tumors	CCR5 antagonist (OB-002)	-	Trial has not yet started	CT.gov
	NCT04721301	Phase I, advanced metastatic colorectal and pancreatic cancer	CCR5 antagonist (Maraviroc)	Nivolumab Ipilimumab (anti-CTLA4)	Results not yet reported	CT.gov
**CXCR1/2**	NCT04477343	Phase I, metastatic PDAC	CXCR1/2 antagonist (SX-682)	Nivolumab	Patient recruitment underway	[207]
	NCT05604560	Phase II, resectable pancreatic cancer	CXCR1/2 antagonist (SX-682)	Tislelizumab (anti-PD1)	Patient recruitment underway	CT.gov
	NCT00851955	PDAC, n = 150	Observational study to correlate CXCR2 receptor/ligand expression and patient outcomes	-	Results not yet reported	CT.gov
**CXCR4/CXCL12**	NCT02179970	Phase I, advanced pancreatic, ovarian, and colorectal cancers, n = 26 (10 PDAC)	CXCR4 antagonist (AMD3100)	-	Well-tolerated, increased T-cell and NK-cell activation and infiltration	[208]
	NCT04177810	Phase II, metastatic PDAC, n = 25	CXCR4 antagonist (AMD3100)	Cemiplimab (anti-PD1)	Results not yet reported	CT.gov
	NCT02826486	Phase IIa, metastatic PDAC, n = 37 (chemotherapy resistant cohort)	CXCR4 antagonist (BL-8040)	Pembrolizumab (anti-PD1)	Well-tolerated, 34.5% disease control rate with 1 partial response, increased CD8^+^ T-cell infiltration	[209]
	NCT02907099	Phase II, metastatic pancreatic cancer n = 20 (15 evaluable for response)	CXCR4 antagonist (BL-8040)	Pembrolizumab	1 partial response, increased cytotoxic CD8^+^ T-cell infiltration post-therapy compared to baseline	[210]
	NCT04543071	Phase II, metastatic treatment-naive pancreatic adenocarcinoma	CXCR4 antagonist (BL-8040)	Cemiplimab Gemcitabine Nab-paclitaxel	Patient recruitment underway	CT.gov
	NCT03168139	Phase I/II, microsatellite stable metastatic colon and pancreatic cancer, n = 20 (9 PDAC)	CXCL12 inhibitor (NOX-A12)	Pembrolizumab	Well-tolerated, 22% disease stabilization in PDAC patients	[211]
	NCT04901741	Phase II, metastatic pancreatic cancer	CXCL12 inhibitor (NOX-A12)	Pembrolizumab and Nanoliposomal Irinotecan or Gemcitabine/Nab-paclitaxel	Trial has not yet started	CT.gov

CT.gov—Information collected from ClinicalTrials.gov, accessed 20 December 2023.

### 7.4. CXCR1/2

To evaluate the effects of CXCR1/2 antagonism in PDAC, SX-682, a CXCR1/2 inhibitor, is currently being tested in a Phase I trial in metastatic PDAC, in combination with anti-PD1, as a maintenance therapy option for unresectable PDAC [207].

### 7.5. CXCR4/CXCL12

The CXCR4/CXCL12 axis holds great promise in PDAC treatment, given its role in cytotoxic T-cell misdirection, metastasis promotion, and the induction of angiogenesis [9,17,105]. In a Phase I trial in metastatic PDAC patients, the administration of the CXCR4 antagonist, AMD3100 (Plerixafor), induced CD8^+^ T-cell infiltration and promoted the activation of T cells and NK cells in patients [208]. Interestingly, within seven days, patients also experienced a significant drop in circulating tumor DNA and serum CXCL8, supporting the possibility of early anti-cancer effects occurring due to treatment [208]. Given these immune-stimulating effects as a single agent, the anti-tumor effects of AMD3100 in combination with anti-PD1 therapy are now being assessed in a Phase II clinical trial (NCT04177810). Another Phase IIa trial using anti-PD1 in combination with BL-8040 (motixafortide), a synthetic peptide antagonist of CXCR4, in metastatic PDAC patients with chemotherapy-resistant disease resulted in a disease control rate (DCR) of 34.5% (n = 29) [209]. Similar to AMD3100, BL-8040 was found to increase CD8^+^ T-cell infiltration [209]. Additionally, intra-tumoral MDSC infiltration and circulatory Tregs were decreased in patients [209]. In a similar Phase II trial combining anti-PD1 therapy with BL-8040 in pancreatic cancer patients, an increase in intra-tumoral CD8^+^ T-cell infiltration post-therapy was also seen [210]. Interestingly, an increase in CD68^+^, PD-L1^+^ macrophages was also noted [210]. Two patients were reported to have a stable disease with 1 partial response (n = 20, 15 evaluable for response) [210]. Additional Phase II trials will assess whether a CXCR4 blockade with BL-8040 improves the anti-tumor efficacy of anti-PD1 treatment (Table 1).

In addition to targeting CXCR4, the anti-tumor effects of NOX-A12 (olaptesed pegol), an L-RNA aptamer inhibitor of CXCL12, have been evaluated in combination with anti-PD1. In a Phase I/II trial assessing NOX-A12 in combination with anti-PD1, featuring both advanced metastatic colorectal (n = 11) and PDAC patients (n = 9), a DCR of 25% was observed [211]. The increased infiltration and migration of T cells towards tumor cores, along with increased cellular activation, was seen in patient biopsy samples [211]. Interestingly, the authors identified a cytokine signature consisting of downregulation in IL-2/IL-16/CXCL10 as associated with tumor resistance [211]. Ongoing trials will evaluate whether a CXCL12 blockade with NOX-A12 can improve overall survival in patients with PDAC (NCT04901741).

## 8. Future Directions

While initial approaches involving modulating the chemokine ligand or receptor expression in PDAC have demonstrated modest efficacy, encouraging biological correlates (increased intra-tumoral CD8^+^ T cells, decreased Tregs) demonstrate that chemokine modulation can have beneficial immune effects. In order to fully translate such efforts into anti-tumor effects, we will require the more precise manipulation of chemokine expression, especially with regard to spatial localization within the PDAC TME. While chemokines do promote tumor progression through various mechanisms, harnessing them for anti-tumor effects will depend on ensuring the appropriate spatial expression within the PDAC TME, ideally at sites of malignant epithelial cells. Given their native role is to stimulate the migration of immune cells, co-opting these properties in order to facilitate the migration of cytotoxic effector cells, such as CD8^+^ T cells and NK cells, towards cancer cells can possibly increase the efficacy of current immunotherapy treatments. Lee et al. discuss an interesting approach towards achieving increased intra-tumoral chemokine elaboration with the creation of an NK-cell–recruiting protein-conjugated antibody (NRP-body) [212]. Here, CXCL16 was conjugated to a mesothelin-targeting antibody, with a furin cleavage site present between CXCL16 and the mesothelin-targeting domain [212]. The furin domain could be cleaved by furin expressed on the surface of pancreatic cancer cells, thus creating a CXCL16 gradient. When mice with orthotopically implanted Panc-1 tumors were then treated with adoptively transferred NK cells and the NRP-body, increased intra-tumoral NK cell accumulation and reduced tumor burden were observed [212]. Thus, this approach demonstrates that artificially induced chemokine gradients can be combined with adoptive cellular therapies, such as CAR-T and CAR-NK, to increase infiltration and anti-tumor effects.

Apart from cellular therapies, it is attractive to consider combining such approaches with monoclonal antibody therapy. Antibodies targeting human epidermal growth factor receptor 2 (HER2), such as trastuzumab, have been successfully utilized to treat HER2-positive cancers, including PDAC in rare cases [213,214]. Apart from direct cytotoxicity, these antibodies modulate indirect mechanisms of tumor cell destruction, most prominently antibody-dependent cellular cytotoxicity (ADCC) [215]. NK cells have been shown to be the main effector immune cell type to modulate ADCC, both in vitro and in vivo [216,217]. While ADCC-promoting antibodies, such as trastuzumab, are FDA-approved and have been shown to induce durable anti-tumor responses in solid tumors, their efficacy in PDAC is limited [218]. However, Beelen et al. demonstrate that the treatment of 3D patient-derived PDAC organoids with either trastuzumab or avelumab (an anti-PD-L1 ADCC-inducing antibody) enhances direct NK cell cytotoxicity, resulting in the destruction of the organoid [219]. Critically, the level of organoid apoptosis was dependent on the effector-to-target ratio, with more NK cells leading to more lysis [219]. This suggests that increasing intra-tumoral NK cell accumulation through chemokine gradients could increase the efficacy of ADCC-promoting antibodies targeting PDAC tumor cell antigens.

In addition to using antibodies to induce intra-tumoral chemokine gradients, therapies that lead to increased cancer cell chemokine signaling can also be utilized to establish intra-tumoral gradients. As discussed earlier, Chibaya et al. found that blocking EZH2 in orthotopic, murine, KPC tumors, treated with MEK and CDK4/6 inhibitors, led to an increase in intra-tumoral CCL2 and CXCL9/10 production [130]. This was then associated with influxes of NK and T cells and anti-tumor responses [130]. Thus, when appropriately targeted, cancer cells themselves can be induced to promote the elaboration of chemokines, such as CXCL9/10, that promote an anti-tumor response. In addition to using small molecules for genetic manipulation/inhibition, viruses are also an interesting technique that have demonstrated promise in inducing chemokine gradients to facilitate immune cell trafficking. While oncolytic viruses can function to preferentially infect cancer cells, leading to lysis due to viral replication, viruses can also be used as vectors to deliver novel immune-modulatory genes that influence immune system action [220]. Kirchhammer et al. demonstrate a novel use of this to enhance the efficacy of IL-12 immunotherapy [221]. Using an adenovirus (AdV5) designed to target tumor cells and induce IL-12 expression in a B16-HER2 orthotopic mouse tumor model, the authors observed that IL-12 anti-tumor efficacy was dependent on CCL5 production by a population of CD49^+^-tissue resident NK cells [221]. However, in tumors lacking these specific CCL5-producing NK cells, the IL-12 therapeutic efficacy could be restored through treatment with an AdV5-CCL5 vector [221]. This combination led to full tumor rejection in 50% of the mice, while AdV5-IL-12 alone did not reject any tumors. Additionally, the combination of AdV5-CCL5 treatment with anti-PD1 in a subcutaneous, B16-HER2 mouse model demonstrated a significant decrease in tumor growth, compared to therapy alone [221]. Thus, utilizing viruses specific to tumors in order to induce chemokine expression represents another promising avenue that can be utilized to create intra-tumoral chemokine gradients that can enhance anti-tumor responses.

Interestingly, cytokine therapy can also be utilized to induce chemokine expression. Cytokine therapy for cancer, most notably IL-2 treatment, has been an FDA-approved modality in melanoma and renal cell carcinoma since 1998 and 1992, respectively [222]. IL-2 therapy works by augmenting the immune response to cancer, activating and inducing effector CD8^+^ T-cell and NK-cell proliferation [222]. However, cytokine therapies appear to also induce the production of intra-tumoral chemokine gradients. Bergamaschi et al. recently documented how heterodimeric IL-15 (hetIL-15), a cytokine known to be involved in the growth and activation of CD8^+^ T cells and NK cells, delays tumor growth in subcutaneous murine MC38 colon carcinoma and TC-1 lung carcinoma models [223]. Flow cytometry analysis of the tumors found increased CD8^+^ T-cell and NK-cell infiltration, as well as significant increases in the secretion of CXCL9 and CXCL10 [223]. However, this secretion appeared to be due to intra-tumoral myeloid cells, not cancer cells [223]. These increases caused by hetIL-15 were IFN-γ-dependent, as increases in CXCL9/10 were not observed in IFN-γ knockout mice [223]. Furthermore, hetIL-15 therapy was associated with an increased frequency of circulating CXCR3^+^ NK and CD8^+^ T cells, suggesting an increased migratory capability in response to CXCL9/10 gradients [223]. Thus, cytokine therapies can potentially be utilized to promote intra-tumoral chemokine production, as well as be potentially combined with site-specific chemokine gradients to increase activated, effector immune cell infiltration.

## 9. Conclusions

Overall, chemokines are critical to PDAC tumorigenesis, progression, therapy resistance, and immune infiltration. Going forward, therapies that are able to take advantage of chemokines’ innate abilities and leverage these abilities to attract immune cells that foster anti-tumor effects can become a powerful tool in the immunotherapy arsenal against PDAC. While initial studies, blocking either chemokines or their cognate receptors (i.e., CCL2, or CXCL12/CXCR4) are promising, they also highlight how chemokine networks in the PDAC TME are still not fully understood and are more nuanced than initially believed. To fully utilize chemokines as agents that promote an anti-tumor immune response, more research will be needed. Critically though, understanding how to spatially localize chemokines to the appropriate locations in the PDAC TME (i.e., at sites of malignant epithelial cells) will be essential in order to utilize their chemotactic abilities to foster an anti-tumor immune response. How this will be accomplished successfully, either using methods such as inducible cellular therapies, antibodies, or oncolytic viruses, still remains to be seen. Moreover, ensuring that these chemokines are attracting the desired cell types (CD8^+^ T cells or NK cells) that mediate anti-tumor responses will need to be refined. While still in the early stages, it is clear that chemokine manipulation has the potential to enhance the standard of care offered by existing therapies to hopefully one day improve outcomes in PDAC patients.

## Data Availability

No new data were created or analyzed in this study. Data sharing is not applicable to this article.
